# Supplementary material for: Polycomb deficiency drives a FOXP2-high aggressive state targetable by epigenetic inhibitors
Source: Nat Commun. 2023 Jan 20;14:336. doi: 10.1038/s41467-023-35784-x (PMC9859827; doi:10.1038/s41467-023-35784-x)
Supplement: Supplementary file 1 — Supplementary Information [file 41467_2023_35784_MOESM1_ESM.pdf]

Supplementary Information

Fan Chen et al.,

Polycomb Deficiency Drives a FOXP2-high Aggressive State Targetable by Epigenetic Inhibitors

Supplementary Tables 1-8

Supplementary Figures and Legends 1-7

Supplementary Table 1: Gene Set Enrichment Analysis for Bulk Tumors

		Ezh2 Het vs WT				*Ezh2* Het vs Null			
		Sorted		Total		Sorted		Total	
	MSigDB Signature Names	ES	negLog(q)	ES	negLog(q)	ES	negLog(q)	ES	negLog(q)
Cold Tumor Gene Signatures	GO_INTERLEUKIN_6_PRODUCTION.grp	-0.648	3.851	-0.735	4.000	-0.517	2.772	-0.304	1.044
	GO_MACROPHAGE_ACTIVATION.grp	-0.658	3.713	-0.767	4.000	-0.634	4.000	-0.308	0.850
	GO_MYELOID_LEUKOCYTE_ACTIVATION.grp	-0.545	2.896	-0.697	4.000	-0.446	3.070	-0.412	3.421
	GO_MYELOID_LEUKOCYTE_MEDIATED_IMMUNITY.grp	-0.553	3.049	-0.693	4.000	-0.417	2.576	-0.452	4.000
	GO_MYELOID_LEUKOCYTE_MIGRATION.grp	-0.677	4.000	-0.701	4.000	-0.489	2.802	-0.485	3.533
	REACTOME_NEUTROPHIL_DEGRANULATION.grp	-0.552	3.040	-0.701	4.000	-0.419	2.575	-0.488	4.000
	GO_NEUTROPHIL_MIGRATION.grp	-0.726	4.000	-0.751	4.000	-0.499	2.240	-0.539	3.882
	GO_GRANULOCYTE_MIGRATION.grp	-0.733	4.000	-0.738	4.000	-0.507	2.420	-0.547	3.811
	BROWN_MYELOID_CELL_DEVELOPMENT_UP.grp	-0.695	4.000	-0.791	4.000	-0.704	4.000	-0.571	4.000
	REACTOME_INTERLEUKIN_10_SIGNALING.grp	-0.811	4.000	-0.850	4.000	-0.747	4.000	-0.741	4.000
	GO_LEUKOTRIENE_METABOLIC_PROCESS.grp	-0.722	2.152	-0.756	3.156	-0.733	2.824	-0.790	4.000
Hot Tumor Gene Signatures	LEE_EARLY_T_LYMPHOCYTE_UP.grp	-0.486	1.394	0.316	0.870	0.502	2.175	-0.267	0.567
	HOEBEKE_LYMPHOID_STEM_CELL_UP.grp	-0.521	1.595	-0.516	1.902	-0.408	1.252	0.468	2.534
	ZHOU_INFLAMMATORY_RESPONSE_LIVE_UP.grp	-0.328	0.315	-0.434	1.378	-0.303	0.833	0.260	0.383
	HADDAD_B_LYMPHOCYTE_PROGENITOR.grp	-0.276	0.093	-0.301	0.197	0.280	0.515	0.460	3.396
	BROWNE_INTERFERON_RESPONSIVE_GENES.grp	-0.485	1.046	-0.468	1.042	0.274	0.106	0.296	0.223

Supplementary Table 2: Gene Enrichment for Principal Component 3 on All Models (Top 40)

GO Term	Description	Enrichment. p	Enrichment. odds	Enrichment. expected	Enrichment. counts	Enrichment. p.adjust
GO:0031012	extracellular matrix	1.60E-14	2.703872	41.16202	94	5.87E-12
GO:0045177	apical part of cell	7.09E-14	2.780568	36.31424	85	2.31E-11
GO:0062023	collagen-containing extracellular matrix	1.81E-13	2.916182	31.20204	76	5.73E-11
GO:0009611	response to wounding	4.18E-12	2.523357	39.66362	86	1.13E-09
GO:0048871	multicellular organismal homeostasis	6.13E-12	2.457509	41.95529	89	1.60E-09
GO:0009615	response to virus	1.19E-11	3.080934	23.09304	59	3.02E-09
GO:0030198	extracellular matrix organization	1.22E-11	3.044034	23.71003	60	3.05E-09
GO:0043062	extracellular structure organization	1.44E-11	3.029359	23.79817	60	3.48E-09
GO:0016324	apical plasma membrane	1.92E-11	2.825989	27.67639	66	4.54E-09
GO:0050900	leukocyte migration	4.28E-11	2.715119	29.43922	68	9.71E-09
GO:0050673	epithelial cell proliferation	4.95E-11	2.489046	36.75495	79	1.10E-08
GO:0030595	leukocyte chemotaxis	1.03E-10	3.275724	18.24526	49	2.11E-08
GO:0051607	defense response to virus	1.63E-10	3.133232	19.65552	51	3.28E-08
GO:0060326	cell chemotaxis	2.30E-10	2.787231	25.38471	60	4.45E-08
GO:0050839	cell adhesion molecule binding	2.40E-10	3.012289	21.06579	53	4.58E-08
GO:0030546	signaling receptor activator activity	2.61E-10	2.304516	42.13157	85	4.93E-08
GO:0048018	receptor ligand activity	3.20E-10	2.305973	41.60273	84	5.99E-08
GO:0042060	wound healing	4.67E-10	2.54032	31.02576	68	8.53E-08
GO:0031589	cell-substrate adhesion	5.28E-10	2.572974	29.79178	66	9.49E-08
GO:0042379	chemokine receptor binding	6.14E-10	6.770754	4.935917	22	1.10E-07
GO:0001667	ameboidal-type cell migration	6.99E-10	2.403898	35.34469	74	1.24E-07
GO:0016323	basolateral plasma membrane	8.40E-10	2.999406	19.91995	50	1.47E-07
GO:0043588	skin development	2.12E-09	2.678616	24.85587	57	3.55E-07
GO:0046977	TAP binding	2.90E-09	93.61202	0.881414	9	4.83E-07
GO:0050678	regulation of epithelial cell proliferation	7.41E-09	2.397907	31.02576	65	1.20E-06
GO:0005902	microvillus	7.71E-09	4.49066	7.932723	27	1.24E-06
GO:0050878	regulation of body fluid levels	8.68E-09	2.424222	29.79178	63	1.38E-06
GO:0090132	epithelium migration	8.95E-09	2.60486	24.5033	55	1.40E-06
GO:0090130	tissue migration	1.16E-08	2.581401	24.67958	55	1.77E-06
GO:0048525	negative regulation of viral process	1.74E-08	4.132851	8.725995	28	2.53E-06
GO:0010631	epithelial cell migration	1.82E-08	2.567568	24.32702	54	2.63E-06
GO:1990868	response to chemokine	2.20E-08	5.228886	5.81733	22	3.07E-06
GO:1990869	cellular response to chemokine	2.20E-08	5.228886	5.81733	22	3.07E-06
GO:0045785	positive regulation of cell adhesion	2.18E-08	2.2257	36.04982	71	3.07E-06
GO:0019221	cytokine-mediated signaling pathway	2.59E-08	2.379486	29.26293	61	3.57E-06
GO:0005903	brush border	2.75E-08	3.498254	11.63466	33	3.75E-06
GO:0043903	regulation of interspecies interactions between organisms	3.06E-08	2.926389	17.01128	42	4.08E-06
GO:0005201	extracellular matrix structural constituent	3.07E-08	3.399201	12.25165	34	4.08E-06
GO:0002064	epithelial cell development	3.17E-08	2.690378	20.80136	48	4.19E-06
GO:0045071	negative regulation of viral genome replication	3.57E-08	5.648252	5.024058	20	4.66E-06

P value is hypergeometric test, P value adjust was Benjamini-Hochberg method

Supplementary Table 3: Gene Set Enrichment Analysis Model Compare

		3D vs 2D		Sorted vs 2D		Sorted vs 3D		Sorted vs Total	
	MSigDB Signature Name	ES	negLog(q)	ES	negLog(q)	ES	negLog(q)	ES	negLog(q)
Chromatin	BENPORATH_EED_TARGETS.grp	0.445	1.580	-0.293	1.224	-0.362	1.826	-0.494	1.603
	BENPORATH_PRC2_TARGETS.grp	0.485	2.013	-0.304	1.096	-0.347	1.277	-0.511	1.718
	PRC2_EED_UP.V1_DN.grp	-0.274	0.932	-0.428	2.863	-0.422	2.227	-0.403	0.635
	PRC2_EED_UP.V1_UP.grp	0.507	2.046	0.356	0.905	-0.217	0.076	-0.244	0.000
	PRC2_EZH2_UP.V1_DN.grp	-0.447	3.228	-0.487	3.543	-0.403	1.791	-0.366	0.355
	PRC2_EZH2_UP.V1_UP.grp	0.526	2.315	0.351	0.915	-0.253	0.246	-0.372	0.405
	GO_CHROMATIN.grp	0.290	0.226	-0.420	3.795	-0.469	4.000	-0.451	1.191
	GO_CHROMATIN_BINDING.grp	0.216	0.054	-0.446	4.051	-0.502	4.000	-0.442	1.052
Cell Cycle	BIOCARTA_CELLCYCLE_PATHWAY.grp	-0.388	0.734	-0.626	2.865	-0.634	2.879	-0.454	0.397
	REACTOME_CELL_CYCLE.grp	-0.505	4.000	-0.510	4.000	-0.411	2.921	-0.185	0.000
	KEGG_CELL_CYCLE.grp	-0.421	2.651	-0.560	4.000	-0.489	3.277	-0.197	0.000
	GO_CELL_CYCLE_DNA_REPLICATION.grp	-0.660	4.000	-0.659	4.000	-0.506	2.511	-0.277	0.000
	GO_DNA_DEPENDENT_DNA_REPLICATION.grp	-0.613	4.000	-0.591	4.000	-0.443	2.398	-0.257	0.000
	GO_DNA_REPLICATION.grp	-0.544	4.000	-0.527	4.000	-0.413	2.393	-0.256	0.000
	KEGG_DNA_REPLICATION.grp	-0.729	4.000	-0.656	4.000	-0.381	0.603	0.211	0.164
	REACTOME_EUKARYOTIC_TRANSLATION_ELONGATION.grp	-0.549	4.000	-0.645	4.000	-0.568	4.000	-0.672	4.000
	HALLMARK_MITOTIC_SPINDLE.grp	-0.356	2.149	-0.555	4.000	-0.544	4.000	-0.393	0.559
	HALLMARK_MYC_TARGETS_V1.grp	-0.642	4.000	-0.537	4.000	-0.321	0.904	-0.198	0.000
	HALLMARK_MYC_TARGETS_V2.grp	-0.725	4.000	-0.543	3.198	-0.299	0.270	0.399	1.958
	REACTOME_MITOTIC_PROMETAPHASE.grp	-0.635	4.000	-0.575	4.000	-0.438	2.623	-0.218	0.000
	REACTOME_MITOTIC_SPINDLE_CHECKPOINT.grp	-0.690	4.000	-0.626	4.000	-0.474	2.646	-0.210	0.000
	HALLMARK_E2F_TARGETS.grp	-0.694	4.000	-0.638	4.000	-0.492	3.438	-0.152	0.000
	HALLMARK_G2M_CHECKPOINT.grp	-0.631	4.000	-0.677	4.000	-0.585	4.000	-0.351	0.292
	BIOCARTA_MCM_PATHWAY.grp	-0.695	3.970	-0.752	4.079	-0.591	1.974	-0.180	0.000
Immunity	BIOCARTA_TOLL_PATHWAY.grp	0.667	2.338	0.397	0.408	-0.423	0.664	0.369	0.907
	HALLMARK_INFLAMMATORY_RESPONSE.grp	0.622	4.000	0.409	1.446	-0.332	0.884	-0.496	1.501
	HALLMARK_INTERFERON_ALPHA_RESPONSE.grp	0.753	4.000	0.503	2.213	-0.458	2.272	-0.261	0.000
	HALLMARK_INTERFERON_GAMMA_RESPONSE.grp	0.690	4.000	0.485	2.865	-0.373	1.464	-0.349	0.267
	REACTOME_IMMUNOREGULATORY_INTERACTIONS_BETWEEN_A_LYMPHOID_AND_A_NON_LYMPHOID_CELL.grp	0.614	2.032	0.675	3.988	0.643	4.000	-0.746	4.000
	REACTOME_INTERFERON_ALPHA_BETA_SIGNALING.grp	0.805	4.000	0.457	1.168	-0.594	3.397	-0.332	0.079
	REACTOME_INTERFERON_GAMMA_SIGANLING.grp	0.707	4.000	0.571	3.176	0.307	0.710	-0.361	0.258
	REACTOME_INTERFERON_SIGNALING.grp	0.609	4.000	0.349	0.893	-0.371	1.448	-0.269	0.000
	REACTOME_COMPLEMENT_CASCADE.grp	0.395	0.168	0.737	4.085	0.829	4.000	-0.463	0.612
	GO_COMPLEMENT_ACTIVATION.grp	0.358	0.144	0.746	4.000	0.820	4.000	-0.481	0.881
	HALLMARK_COMPLEMENT.grp	0.474	1.735	0.396	1.341	0.288	0.818	-0.538	2.024
	KEGG_ANTIGEN_PROCESSING_AND_PRESENTATION.grp	0.698	3.924	0.655	3.714	0.612	4.000	-0.450	0.643
	GO_HUMORAL_IMMUNE_RESPONSE.grp	0.600	3.324	0.672	4.000	0.558	4.000	-0.381	0.423
	GO_REGULATION_OF_HUMORAL_IMMUNE_RESPONSE.grp	0.569	1.281	0.780	4.000	0.751	4.000	-0.490	0.982
	HALLMARK_ALLOGRAFT_REJECTION.grp	0.464	1.439	0.447	2.053	0.351	1.469	-0.666	4.000
	HALLMARK_COAGULATION.grp	0.385	0.627	0.484	2.202	0.433	2.420	-0.339	0.190
Lung Lineage	MOUSE_LUNG_LINEAGE.grp	0.925	4.000	0.880	4.000	0.616	4.000	0.569	4.000
	REACTOME_KERATINIZATION.grp	0.767	4.000	0.446	1.085	-0.497	2.185	0.377	1.413
	REACTOME_SURFACTANT_METABOLISM.grp	0.815	4.000	0.798	4.000	0.598	2.429	-0.522	0.872
	BIOCARTA_KERATINOCYTE_PATHWAY.grp	0.620	2.523	-0.252	0.098	-0.476	1.693	-0.403	0.382
	GO_AXONEME_ASSEMBLY.grp	0.367	0.173	0.723	4.000	0.707	4.000	0.756	4.000
	GO_DYNEIN_COMPLEX.grp	0.351	0.175	0.702	4.000	0.675	4.000	0.541	4.000
	KEGG_DRUG_METABOLISM_CYTOCHROME_P450.grp	0.776	4.000	0.687	4.126	0.375	0.855	0.515	2.530
	GO_CELL_FATE_COMMITMENT.grp	0.455	1.402	-0.404	2.071	-0.538	4.000	-0.547	2.113
	GO_KERATINIZATION.grp	0.759	4.000	0.398	0.857	-0.525	2.938	0.419	1.924
	GO_LUNG_ALVEOLUS_DEVELOPMENT.grp	0.684	3.309	0.415	0.776	-0.509	1.841	-0.541	1.352
	GO_LUNG_EPITHELIUM_DEVELOPMENT.grp	0.609	1.991	0.453	0.940	-0.332	0.255	-0.460	0.610
	GO_MOTILE_CILIUM.grp	0.289	0.147	0.597	4.000	0.567	4.000	0.536	4.000
	GO_POSITIVE_REGULATION_OF_EPITHELIAL_CELL_PROLIFERATION_INVOLVED_IN_LUNG_MORPHOGENESIS.grp	-	-	0.763	1.308	-	-	-0.521	0.097
	GO_EPITHELIAL_CELL_PROLIFERATION_INVOLVED_IN_LUNG_MORPHOGENESIS.grp	0.632	0.986	0.713	1.658	0.694	1.800	-0.255	0.000
	GO_POSITIVE_REGULATION_OF_EPITHELIAL_CELL_PROLIFERATION.grp	0.428	1.173	0.292	0.450	-0.305	0.621	-0.436	0.884

Supplementary Table 4: Gene Set Enrichment Analysis Between *Ezh2* Genotypes

		Ezh2 Het vs WT						*Ezh2* Null vs Het						*Ezh2* Null vs WT					
		2D		3D		Sorted		2D		3D		Sorted		2D		3D		Sorted	
	MSigDB Signature Name	ES	negLog(q)	ES	negLog(q)	ES	negLog(q)	ES	negLog(q)	ES	negLog(q)	ES	negLog(q)	ES	negLog(q)	ES	negLog(q)	ES	negLog(q)
Histone and DNA Methylation	GO_HISTONE_METHYLTRANSFERASE_COMPLEX.grp	0.122	0.000	0.198	0.000	0.546	2.903	-0.255	0.177	-0.221	0.022	-0.386	0.966	-0.322	0.400	0.123	0.000	0.275	0.322
	GO_HISTONE_LYSINE_N_METHYLTRANSFERASE_ACTIVITY.grp	0.135	0.000	0.309	0.000	0.515	1.776	-0.421	0.848	-0.266	0.027	-0.454	1.078	-0.436	0.775	0.194	0.000	-0.199	0.014
	REACTOME_PKMTS_METHYLATE_HISTONE_LYSINES.grp	0.214	0.007	0.291	0.000	0.505	1.751	-0.508	1.431	-0.345	0.427	-0.516	1.721	-0.505	1.188	-0.175	0.000	-0.293	0.065
	GO_PCG_PROTEIN_COMPLEX.grp	0.356	0.464	0.340	0.062	0.616	2.911	-0.372	0.614	-0.272	0.038	-0.554	2.046	-0.263	0.015	0.297	0.000	-0.184	0.016
	GO_HISTONE_METHYLATION.grp	0.194	0.011	0.212	0.000	0.444	2.078	-0.203	0.101	-0.175	0.000	-0.383	1.144	0.256	0.015	0.193	0.000	0.204	0.061
	HOQUE_METHYLATED_IN_CANCER.grp	-0.365	0.409	-0.702	2.998	-0.517	1.032	0.608	1.453	-0.417	0.716	-0.316	0.180	0.649	1.614	-0.448	0.808	-0.473	0.801
	WANG_METHYLATED_IN_BREAST_CANCER.grp	0.530	1.347	-0.461	0.666	0.438	0.891	-0.419	0.748	0.372	0.141	0.426	0.812	0.443	0.297	-0.348	0.179	0.609	2.780
	GO_REGULATION_OF_HISTONE_METHYLATION.grp	0.308	0.307	0.250	0.000	0.413	1.045	0.193	0.002	-0.153	0.001	-0.276	0.141	0.396	0.248	0.225	0.000	0.294	0.316
	GO_COVALENT_CHROMATIN_MODIFICATION.grp	0.221	0.234	0.159	0.000	0.392	1.849	0.184	0.000	-0.191	0.031	-0.283	0.628	0.216	0.005	0.139	0.000	0.207	0.270
	GO_HISTONE_H3_K9_MODIFICATION.grp	0.232	0.017	0.351	0.087	0.288	0.074	0.407	0.214	-0.195	0.002	-0.220	0.009	0.545	0.842	0.325	0.000	0.387	0.638
Polycomb Gene Targets	KAMMINGA_EZH2_TARGETS.grp	0.582	2.238	0.270	0.000	-0.169	0.022	-0.339	0.420	-0.259	0.032	-0.634	2.722	0.490	0.486	-0.169	0.001	-0.634	2.338
	BRIDEAU_IMPRINTED_GENES.grp	-0.468	0.873	-0.516	1.247	0.412	0.913	0.622	1.570	0.742	4.000	0.562	2.560	0.576	1.105	0.684	3.031	0.571	3.071
	PASINI_SUZ12_TARGETS_DN.grp	0.293	0.730	-0.529	3.251	-0.295	0.162	0.369	0.375	-0.283	0.602	0.264	0.466	0.466	1.083	-0.496	3.499	0.255	0.460
	MEISSNER_NPC_HCP_WITH_H3K4ME3_AND_H3K27ME3.grp	0.584	2.888	0.375	0.224	-0.379	0.397	0.499	0.915	0.429	0.698	-0.325	0.453	0.616	1.906	0.441	0.789	0.335	0.631
	MEISSNER_BRAIN_HCP_WITH_H3K27ME3.grp	-0.363	0.614	-0.393	0.636	-0.424	0.709	0.702	3.564	0.504	1.225	0.436	1.520	0.719	4.000	-0.346	0.493	-0.403	0.756
	BENPORATH_EED_TARGETS.grp	0.344	1.255	-0.374	1.111	-0.457	1.490	0.641	3.827	0.341	0.566	-0.289	0.728	0.637	4.000	-0.374	1.473	-0.453	1.720
	BENPORATH_SUZ12_TARGETS.grp	0.343	1.255	-0.384	1.198	-0.484	1.883	0.593	3.084	0.400	0.980	-0.311	0.963	0.572	2.673	0.345	0.692	-0.499	2.326
	BENPORATH_ES_WITH_H3K27ME3.grp	-0.363	1.145	-0.455	2.165	-0.495	2.046	0.643	3.631	0.436	1.296	-0.292	0.784	0.626	4.000	-0.361	1.321	-0.469	1.899
	MIKKELSEN_MCV6_HCP_WITH_H3K27ME3.grp	-0.378	0.816	-0.466	1.422	-0.510	1.833	0.607	2.490	0.624	4.000	0.322	0.722	0.503	1.064	0.469	1.469	-0.461	1.374
	MIKKELSEN_IPS_WITH_HCP_H3K27ME3.grp	-0.482	0.594	-0.659	1.843	-0.515	0.637	0.878	4.000	0.566	0.936	0.409	0.412	0.896	4.000	-0.563	1.275	-0.509	0.769
	MIKKELSEN_NPC_HCP_WITH_H3K27ME3.grp	0.478	2.055	-0.460	1.298	-0.519	1.876	0.667	3.962	0.588	3.091	0.356	0.987	0.690	4.000	0.508	1.842	-0.445	1.186
	ACEVEDO_LIVER_CANCER_WITH_H3K27ME3_UP.grp	0.275	0.369	-0.324	0.385	-0.519	2.052	0.622	2.974	0.354	0.448	0.405	1.706	0.620	2.653	-0.324	0.565	-0.383	0.770
	BMI1_DN.V1_UP.grp	0.412	1.359	-0.553	2.668	-0.536	2.122	0.473	0.933	-0.471	1.951	0.376	1.155	0.489	0.965	-0.596	3.903	-0.448	1.308
	MEISSNER_NPC_HCP_WITH_H3K27ME3.grp			-0.347	0.022	-0.547	0.728	0.856	3.786	0.661	1.070	-0.468	0.548	0.839	3.032	0.616	1.121	-0.670	1.529
	BENPORATH_PRC2_TARGETS.grp	0.380	1.349	-0.447	1.511	-0.556	2.847	0.661	3.689	0.419	0.975	0.318	0.841	0.636	3.624	0.363	0.680	-0.523	2.389
	MIKKELSEN_MEF_HCP_WITH_H3K27ME3.grp	-0.417	1.184	-0.476	1.681	-0.585	3.575	0.370	0.279	0.622	4.000	0.358	1.197	-0.395	1.009	0.452	1.366	-0.479	1.728
HOX Genes	GO_ANTERIOR_POSTERIOR_PATTERN_SPECIFICATION.grp	-0.333	0.553	-0.289	0.135	-0.282	0.081	0.769	4.000	-0.346	0.741	-0.302	0.461	0.776	4.000	0.292	0.015	-0.390	0.748
	GO_EMBRYONIC_ORGAN_MORPHOGENESIS.grp	0.394	1.380	-0.414	1.120	-0.342	0.334	0.471	0.993	-0.413	1.488	-0.364	1.131	0.540	1.606	-0.464	2.180	-0.448	1.308
	GO_EMBRYONIC_SKELETAL_SYSTEM_DEVELOPMENT.grp	0.338	0.597	-0.477	1.257	0.285	0.131	0.815	4.000	-0.421	1.039	-0.582	2.968	0.840	4.000	-0.612	3.591	-0.561	2.139
	GO_SKELETAL_SYSTEM_MORPHOGENESIS.grp	-0.321	0.596	-0.501	2.019	-0.332	0.256	0.721	4.000	-0.396	1.255	-0.461	2.096	0.711	4.000	-0.616	4.000	-0.522	2.162
	REACTOME_ACTIVATION_OF_ANTERIOR_HOX_GENES_IN_HINDBRAIN_	0.333	0.457	0.193	0.000	0.421	1.122	0.558	1.451	0.287	0.049	-0.475	1.583	0.694	3.539	-0.251	0.024	-0.328	0.181
EMT	AIGNER_ZEB1_TARGETS.grp	0.562	1.583	-0.475	0.810	-0.305	0.063	-0.714	4.000	-0.365	0.381	0.334	0.283	-0.664	2.435	-0.445	0.808	0.334	0.318
	ANASTASSIOU_MULTICANCER_INVASIVENESS_SIGNATURE.grp	-0.369	0.408	-0.819	4.000	-0.705	3.841	0.811	3.786	0.525	0.743	-0.643	3.516	0.809	4.000	-0.788	4.000	-0.802	4.000
	GO_CLUSTER_OF_ACTIN_BASED_CELL_PROJECTIONS.grp	-0.380	0.801	0.340	0.259	-0.346	0.286	0.304	0.041	0.538	1.887	0.290	0.367	-0.381	0.794	0.581	3.128	-0.333	0.333
	GO_COMPLEX_OF_COLLAGEN_TRIMERS.grp					-0.740	2.604	0.723	1.649			0.367	0.214	0.829	2.404			-0.685	2.018
	GO_EXTRACELLULAR_MATRIX_STRUCTURAL_CONSTITUENT.grp	0.356	0.712	-0.672	3.759	-0.644	3.905	0.577	1.745	0.397	0.483	-0.462	1.911	0.613	2.033	-0.575	3.033	-0.691	4.000
	HALLMARK_EPITHELIAL_MESENCHYMAL_TRANSITION.grp	0.313	0.677	-0.694	4.000	-0.505	1.877	0.613	2.948	0.379	0.563	-0.425	1.805	0.633	3.272	-0.627	4.000	-0.565	3.151
	HOLLERN_EMT_BREAST_TUMOR_UP.grp	-0.415	1.145	-0.643	4.000	-0.638	3.865	0.585	2.084	0.363	0.441	0.508	2.966	0.499	1.075	-0.506	2.550	-0.372	0.621
	JECHLINGER_EPITHELIAL_TO_MESENCHYMAL_TRANSITION_UP.grp	-0.412	0.774	-0.641	2.964	-0.653	3.598	0.647	2.454	-0.451	1.071	-0.314	0.314	0.587	1.316	-0.592	2.890	-0.659	4.321
	MISHRA_CARCINOMA_ASSOCIATED_FIBROBLAST_UP.grp					-0.505	0.635					-0.446	0.635					-0.533	0.887
	ONDER_CDH1_TARGETS_2_UP.grp	-0.337	0.786	-0.483	1.898	-0.457	1.377	0.669	3.740	0.435	1.055	-0.362	1.144	0.695	4.000	-0.360	0.977	-0.514	2.273
	NABA_COLLAGENS.grp	0.382	0.284	-0.630	1.357	-0.538	0.983	0.750	2.502			-0.371	0.317	0.810	2.926	-0.704	2.245	-0.581	1.380
	NABA_ECM_GLYCOPROTEINS.grp	0.363	0.785	-0.620	3.223	-0.698	4.000	0.586	1.911	0.447	0.844	-0.454	1.893	0.611	2.012	-0.499	2.036	-0.743	4.000
	PEDERSEN_METASTASIS_BY_ERBB2_ISOFORM_1.grp	-0.430	0.794	0.353	0.104	0.504	1.665	0.430	0.283	0.355	0.162	0.432	0.993	0.374	0.117	0.390	0.266	0.677	4.000
	REACTOME_COLLAGEN_BIOSYNTHESIS_AND_MODIFYING_ENZYMES.grp	-0.340	0.297	-0.615	1.852	-0.411	0.369	0.701	2.737	-0.488	1.021	-0.395	0.724	0.719	2.881	-0.657	3.011	-0.523	1.369
	REACTOME_COLLAGEN_CHAIN_TRIMERIZATION.grp	0.382	0.311	-0.630	1.288	-0.538	1.042	0.750	2.462			-0.371	0.319	0.810	2.843	-0.704	2.376	-0.581	1.531
	REACTOME_COLLAGEN_FORMATION.grp	-0.353	0.521	-0.643	3.196	-0.517	1.416	0.636	2.347	-0.503	1.393	-0.329	0.455	0.643	2.066	-0.712	4.000	-0.523	1.609
	REACTOME_EXTRACELLULAR_MATRIX_ORGANIZATION.grp	-0.314	0.595	-0.577	3.347	-0.607	3.892	0.528	1.645	-0.406	1.408	-0.350	1.069	0.532	1.609	-0.582	4.000	-0.625	4.000
	SARRIO_EPITHELIAL_MESENCHYMAL_TRANSITION_UP.grp	0.514	2.411	-0.217	0.013	-0.258	0.061	-0.266	0.423	-0.315	0.648	-0.360	1.061	0.399	0.438	-0.305	0.480	-0.424	1.051
	WU_CELL_MIGRATION.grp	0.343	0.916	-0.470	1.646	-0.444	1.090	-0.359	0.998	-0.326	0.719	0.411	1.711	0.375	0.329	-0.483	2.387	0.290	0.580
Immunity	REACTOME_INTERLEUKIN_10_SIGNALING.grp	-0.529	0.965	-0.623	1.629	-0.811	4.000	-0.611	1.448	-0.690	2.803	0.747	4.000	-0.585	1.161	-0.819	4.000	-0.572	1.511
	GO_INTERLEUKIN_6_PRODUCTION.grp	0.355	0.821	-0.400	0.820	-0.648	3.851	0.324	0.082	-0.471	1.841	0.517	2.772	0.396	0.327	-0.629	4.000	-0.440	1.089
	ZHANG_INTERFERON_RESPONSE.grp			0.491	0.246	-0.615	1.375	0.836	2.685	-0.847	4.000	0.262	0.003	0.830	2.280	-0.779	3.747	-0.554	1.020
	HALLMARK_INTERFERON_ALPHA_RESPONSE.grp	-0.409	0.910	0.346	0.205	-0.453	1.004	0.685	3.990	-0.736	4.000	-0.202	0.009	0.642	2.651	-0.674	4.000	-0.435	1.000
	BROWNE_INTERFERON_RESPONSIVE_GENES.grp	-0.357	0.426	0.455	0.577	-0.485	1.046	0.714	3.563	-0.719	4.000	-0.274	0.106	0.686	2.316	-0.669	3.875	-0.469	0.995
	BOSCO_INTERFERON_INDUCED_ANTIVIRAL_MODULE.grp	-0.389	0.586	0.337	0.095	-0.625	2.778	0.705	3.062	-0.695	4.000	0.350	0.602	0.675	2.198	-0.534	1.845	-0.609	2.725
	GO_RESPONSE_TO_TYPE_I_INTERFERON.grp	0.312	0.407	0.281	0.015	-0.369	0.343	0.615	2.017	-0.694	4.000	0.245	0.066	0.688	2.982	-0.638	4.000	-0.320	0.180
	GO_DEFENSE_RESPONSE_TO_VIRUS.grp	0.228	0.150	-0.252	0.066	-0.450	1.236	0.541	1.798	-0.546	4.000	0.279	0.463	0.568	2.057	-0.537	3.610	-0.367	0.685
	GO_RESPONSE_TO_INTERFERON_GAMMA.grp	-0.327	0.557	-0.418	1.113	-0.513	1.869	0.532	1.483	-0.506	2.829	0.276	0.372	0.535	1.358	-0.571	3.954	-0.441	1.210
	HALLMARK_TNFA_SIGNALING_VIA_NFKB.grp	0.274	0.453	-0.495	2.289	0.432	2.319	-0.359	1.011	-0.411	1.508	0.281	0.466	-0.393	1.187	-0.512	3.420	0.481	3.579
	TIAN_TNF_SIGNALING_VIA_NFKB.grp	0.422	0.647	-0.632	1.478	-0.368	0.096	-0.788	4.000	-0.680	2.548	-0.306	0.074	-0.764	4.000	-0.796	3.978	0.343	0.329
	RADAEVA_RESPONSE_TO_IFNA1_UP.grp	-0.398	0.565	0.362	0.114	-0.438	0.534	0.762	4.016	-0.678	3.453	-0.290	0.099	0.662	1.744	-0.665	3.437	-0.494	0.963
	HECKER_IFNB1_TARGETS.grp	-0.604	1.867	-0.416	0.668	-0.556	1.878	0.743	3.899	-0.671	4.000	-0.321	0.362	0.645	1.698	-0.597	2.941	-0.587	2.389
	HALLMARK_INTERFERON_GAMMA_RESPONSE.grp	-0.320	0.588	-0.287	0.197	-0.497	1.769	0.536	1.650	-0.671	4.000	0.299	0.629	0.491	1.076	-0.651	4.000	-0.447	1.307
	SEKI_INFLAMMATORY_RESPONSE_LPS_UP.grp	-0.343	0.424	-0.494	1.435	-0.473	1.081	-0.373	0.736	-0.644	4.000	0.501	2.048	-0.477	1.143	-0.662	4.000	-0.306	0.137
	HALLMARK_INFLAMMATORY_RESPONSE.grp	0.353	0.931	-0.427	1.178	-0.641	3.854	0.304	0.058	-0.555	4.000	0.510	3.091	0.416	0.467	-0.609	4.000	-0.483	1.702
	GO_LEUKOCYTE_MIGRATION.grp	0.347	1.144	-0.429	1.425	-0.652	4.000	0.390	0.466	-0.416	1.875	0.437	2.634	0.463	1.058	-0.466	2.565	-0.549	3.145
	HALLMARK_IL6_JAK_STAT3_SIGNALING.grp	0.393	0.904	-0.406	0.615	-0.535	1.774	0.351	0.106	-0.459	1.313	0.439	1.507	0.442	0.433	-0.532	2.219	-0.429	0.886
	KEGG_CYTOKINE_CYTOKINE_RECEPTOR_INTERACTION.grp	0.395	1.204	-0.560	2.797	-0.641	3.835	-0.342	0.768	-0.514	2.807	0.410	1.624	0.410	0.432	-0.643	4.000	-0.571	2.861
	ALTEMEIER_RESPONSE_TO_LPS_WITH_MECHANICAL_VENTILATION.grp	-0.381	0.805	-0.474	1.289	-0.616	3.262	0.436	0.504	-0.626	4.000	0.519	2.823	0.446	0.426	-0.667	4.000	-0.440	1.089
	DER_IFN_ALPHA_RESPONSE_UP.grp	-0.256	0.064	-0.389	0.594	-0.284	0.062	0.541	1.165	-0.623	4.000	-0.298	0.294	0.472	0.537	-0.651	3.929	-0.340	0.272

Supplementary Table 5: H3K27me3 Peaks Shared by *Ezh2* Het vs *Ezh2* WT or *Ezh2* Null

1568 Genes Associated with 1082 Conserved H3K27me3 Peaks in All Three *Ezh2* Genotypes
Gene Families of Interest are Highlighted in Yellow

Aasdh	Bsx	Cyp26b1	Fbxo2	Grrp1	Kif1a	Neurog2	Pknox2	Rtn4rl2	Sptlc1	Vgf
Aatk	Btaf1	Cyp46a1	Fbxo44	Gsc	Kif26a	Nfam1	Pkp1	Runx3	Srrm3	Vgll2
Abcb5	Btrc	Cys1	Fbxw4	Gsx1	Kif26b	Nfatc1	Plcl1	Rxfp3	Srxn1	Vim
Abcc4	C1qa	Cyth4	Fcnb	Gsx2	Kirrel2	Nfatc2	Plekhd1	Rybp	Sstr2	Vipr1
Abcd4	C1ql3	Cytip	Fem1b	Gtdc1	Kirrel3	Nfe2l3	Plin5	S1pr3	Sstr5	Vsx2
Abcg4	C1ql4	Cyyr1	Fev	H13	Kit	Ngfr	Plk5	S1pr5	Ssx2ip	Vwa5b2
Abhd5	C1qtnf4	D430041D0	Fez1	H1fx	Klf15	Nhlh2	Pltp	Sall2	St3gal4	Vwa8
Abt1	C2cd4a	D630044L2	Fezf2	H2afy	Klhdc7a	Nhsl2	Plvap	Sall3	Stac2	Wdpcp
Abtb1	Cabp1	Daam1	Fgf15	H2afy2	Klhl15	Nkain3	Plxdc1	Sall4	Stard4	Wdr24
Acacb	Cabp7	Dact1	Fgf16	H2-Q1	Klhl29	Nkd1	Plxna4	Satb2	Stk17b	Wdr36
Acbd6	Cacna1c	Dagla	Fgf20	H6pd	Klhl30	Nkd2	Plxnc1	Sbk1	Stk33	Wdr41
Accsl	Cacna1g	Dbn1	Fgf3	Hand1	Klhl42	Nkiras2	Pmch	Scaf11	Stum	Wdr76
Acot7	Cacna1h	Dbndd1	Fgf4	Hand2	Krt1	Nkpd1	Pmp22	Scarf2	Stx17	Wfdc18
Acr	Cacna1i	Dbx1	Fgf5	Hapln2	Krt5	Nkx1-1	Pnmal2	Scn1b	Stx8	Wfdc21
Acsf3	Cacna1s	Dclk2	Fgf8	Hapln4	Krt71	Nkx1-2	Podn	Scn4b	Sulf2	Wipf1
Actr5	Cacna2d2	Dclk3	Fgf9	Has2	Lamc3	Nkx2-1	Podxl2	Scn5a	Sult5a1	Wipf3
Acvr1c	Cacnb4	Ddn	Fgfr1	Hck	Laptm5	Nkx2-2	Pofut2	Scrg1	Sv2c	Wiz
Acvrl1	Calcoco1	Ddx39	Fhod3	Hcn4	Lbx1	Nkx2-3	Pola1	Scrt1	Syndig1l	Wnk2
Adam11	Camk2b	Deaf1	Fibcd1	Hcrtr2	Lef1	Nkx2-4	Polg	Scrt2	Synm	Wnt1
Adam33	Camk2d	Dedd2	Fli1	Hdac4	Lemd1	Nkx2-5	Poll	Scube2	Sypl2	Wnt10a
Adamts1	Camk4	Dennd1a	Flnc	Hdgfl3	Letm2	Nkx2-6	Poln	Scube3	Syt17	Wnt10b
Adamts14	Car15	Dennd2a	Flrt1	Hdhd5	Lgi3	Nkx3-1	Polr2m	Sdc3	Syt2	Wnt11
Adamts8	Car4	Det1	Flt1	Hectd2	Lgr5	Nkx3-2	Pou2f2	Sdr16c6	Syt6	Wnt2
Adamtsl5	Car7	Dgcr14	Fmn1	Hecw2	Lgr6	Nkx6-1	Pou3f1	Sdsl	Syt7	Wnt2b
Adarb1	Caskin1	Dgkg	Fndc5	Helt	Lhx1	Nkx6-2	Pou3f2	Sec13	Tab2	Wnt3
Adcy5	Casq2	Dgkh	Foxa2	Hes2	Lhx2	Nkx6-3	Pou3f3	Sec22a	Tacr3	Wnt3a
Adcy7	Cav3	Dgki	Foxb1	Hes3	Lhx3	Nlrx1	Pou4f1	Sec22c	Taf7	Wnt4
Add3	Cbfa2t3	Dgkz	Foxb2	Hes5	Lhx4	Nnmt	Pou4f2	Sec31b	Tal1	Wnt5a
Adgra2	Cbll1	Dhdds	Foxd1	Hes7	Lhx5	Nog	Pou5f2	Sec61a1	Tas1r2	Wnt5b
Adgrb1	Cbln1	Dhh	Foxd2	Hey1	Lhx6	Nol4	Ppargc1a	Sema5b	Tax1bp1	Wnt7a
Adgrb2	Cbx4	Dhx15	Foxd4	Hey2	Lhx9	Nol4l	Ppif	Sema6a	Tbata	Wnt8b
Adgrl1	Cbx5	Dio3	Foxe1	Hfe2	Lilr4b	Notum	Ppip5k1	Sema6b	Tbc1d16	Wnt9b
Adora2a	Cbx8	Dirc2	Foxe3	Hhip	Lin28a	Nova2	Ppm1e	Sema6c	Tbc1d24	Wrap73
Adra2a	Cc2d2b	Disc1	Foxf1	Hhipl1	Lingo1	Npas1	Ppm1j	Sept3	Tbc1d9	Wt1
Adra2b	Ccbe1	Dlgap2	Foxf2	Hic1	Lingo3	Nphp3	Ppm1n	Sept9	Tbca	Xirp2
Adrb1	Ccdc124	Dlgap3	Foxi3	Hif1an	Lix1l	Nphs1	Ppp1r37	Serpine2	Tbkbp1	Xkr7
Aebp1	Ccdc136	Dlgap4	Foxj2	Higd1b	Lman1	Npm1	Ppp1r3g	Serpinh1	Tbpl1	Ybx2
Aen	Ccdc155	Dlk2	Foxl1	Hivep3	Lmo2	Nppc	Ppp2r2c	Setx	Tbx1	Ythdf1
Agap2	Ccdc167	Dlx1	Foxl2	Hk3	Lmx1a	Npr1	Ppp2r5c	Sez6	Tbx15	Ywhae
Agtrap	Ccdc177	Dlx2	Foxn3	Hlcs	Lmx1b	Npr2	Ppy	Sfmbt2	Tbx2	Zbtb16
Ahi1	Ccdc182	Dlx3	Foxn4	Hlf	Lor	Nptx1	Prcd	Sfrp2	Tbx20	Zbtb40
Ak2	Ccdc188	Dlx4	Foxo6	Hlx	Lpar3	Nptxr	Prdm12	Sfrp5	Tbx21	Zbtb43
Ak4	Ccdc40	Dlx5	Foxs1	Hmx1	Lpcat2b	Npvf	Prdm13	Sh2d1b1	Tbx4	Zbtb44
Akap10	Ccin	Dlx6	Fras1	Hmx2	Lpin2	Npy	Prdm14	Sh2d1b2	Tcea1	Zbtb46
Akap2	Cck	Dmbx1	Frmd4b	Hoxa13	Lpo	Nr2e1	Prdm6	Sh2d4a	Tcerg1l	Zbtb7c
Aldh1a2	Ccm2l	Dmrt3	Frmd5	Hoxa3	Lrg1	Nr2f1	Prdm8	Sh3pxd2a	Tcf20	Zc3h8
Aldh1l1	Ccna1	Dmrta1	Frmpd1	Hoxa6	Lrmp	Nr2f2	Prdx2	Sh3rf3	Tcf21	Zcchc13
Alg3	Ccnd2	Dmrta2	Frmpd3	Hoxb13	Lrp2	Nr4a3	Prex1	Shank1	Tchh	Zcchc24

Alkal2	Ccnh	Dmxl2	Frzb	Hoxb4	Lrrc10b	Nr5a1	Prkcsh	Shank3	Tchhl1	Zdbf2
Alox12b	Ccnj	Dnaic1	Fstl4	Hoxb5	Lrrc4b	Nr5a2	Prkra	Shc3	Tcte1	Zdhhc14
Alox8	Ccr4	Dnajc19	Fxyd2	Hoxc13	Lrrc75b	Nrcam	Prlhr	She	Tectb	Zdhhc7
Alpk1	Cd207	Dnajc22	Fxyd6	Hoxc4	Lrrn2	Nrg2	Prmt6	Shisa3	Tesc	Zeb1
Alx1	Cd248	Dnajc5g	Fxyd7	Hoxc8	Lrrn4	Nrip3	Prmt8	Shisa8	Tex29	Zeb2
Alx3	Cd3d	Dnase2a	Fzd3	Hoxd13	Lsm14b	Nrl	Prok2	Shmt2	Tfap2a	Zfand2a
Alx4	Cd3g	Dntt	Fzd9	Hoxd8	Ltbp4	Nrxn2	Prox1	Shox2	Tfap2b	Zfhx4
Amer2	Cdc20b	Doc2b	Gabpb2	Hoxd9	Ly6h	Nsf	Prr7	Sim1	Tfap2e	Zfp11
Amfr	Cdca2	Dock3	Gad1	Hpcal4	Lyzl4	Nsmaf	Prrt4	Sim2	Tgfbr1	Zfp281
Ank1	Cdh22	Dpf3	Galnt16	Hps1	Lzts1	Nt5c1a	Prrx1	Sipa1l2	Them7	Zfp322a
Ank2	Cdh23	Dpysl5	Galr2	Hpse2	Mab21l2	Nt5c1b	Prrxl1	Six1	Tle3	Zfp326
Ankrd26	Cdk18	Draxin	Galr3	Hrh2	Macf1	Nt5c2	Prss12	Six2	Tlr2	Zfp385a
Ankrd27	Cdk5r2	Drd4	Garem2	Hrh3	Mad1l1	Ntn1	Prune2	Six3	Tlx1	Zfp385c
Ankrd33b	Cdk5rap3	Dscaml1	Gars	Hrh4	Maf	Ntng1	Psapl1	Six6	Tlx2	Zfp423
Ankrd34b	Cdx1	Dusp10	Gas7	Hrk	Mafa	Ntng2	Psd	Skor1	Tlx3	Zfp454
Ankrd53	Cebpe	Dync1li1	Gas8	Hs3st3a1	Map1a	Ntrk1	Psd2	Slc12a7	Tm6sf1	Zfp462
Ankrd54	Cecr2	Dysf	Gata2	Hs3st3b1	Map1b	Ntsr1	Psmb2	Slc13a5	Tmcc2	Zfp503
Ankrd63	Celsr3	E030025P	Gata4	Hs3st6	Map2k3	Nuak2	Psmb7	Slc16a10	Tmeff2	Zfp518a
Anks1b	Cemip	Ebf1	Gata5	Hsd17b12	Map6	Numbl	Psmc1	Slc16a9	Tmem104	Zfp521
Anxa3	Cep120	Ebf2	Gbx1	Hspb1	Mapk4	Nup214	Psmf1	Slc17a7	Tmem108	Zfp523
Ap1b1	Cep135	Ebf3	Gbx2	Htatip2	Mapre3	Nupl2	Ptch1	Slc1a2	Tmem115	Zfp593
Ap1g2	Cep164	Ebf4	Gcnt1	Htra1	March4	Nxph1	Ptchd1	Slc1a7	Tmem132b	Zfp64
Ap1s1	Cep72	Ecel1	Gcnt3	Hydin	Mast1	Nxph3	Ptger3	Slc22a12	Tmem145	Zfp804a
Ap3b1	Cfap44	Edn2	Gdf10	Ice2	Matn1	Nxph4	Ptgfr	Slc24a4	Tmem150c	Zfpm1
Apba1	Cfap65	Ednrb	Gdf2	Ifi44l	Mbip	Oaz1	Ptgis	Slc25a18	Tmem151a	Zfyve28
Apcdd1	Chchd4	Efcab11	Gdf6	Ifngr1	Mcam	Obscn	Pth1r	Slc25a37	Tmem151b	Zic3
Apeh	Chd2	Efcc1	Gdf7	Igdcc3	Mcidas	Obsl1	Pth2	Slc26a4	Tmem160	Zpbp2
Aqp4	Chmp1b	Efhd1	Gdi2	Igf1	Mcrs1	Odf3l1	Pthlh	Slc26a6	Tmem161b	Zranb2
Aqp6	Chn1	Efna1	Gfi1	Igf1r	Mdga1	Ofcc1	Ptn	Slc2a13	Tmem163	Zswim2
Arhgap12	Chpf	Efna3	Gfra1	Igf2	Med13l	Olfm2	Ptpn5	Slc2a2	Tmem174	Zswim5
Arhgap20	Chrdl2	Efr3b	Gfra4	Igf2bp1	Med31	Olig1	Ptpru	Slc2a3	Tmem18	
Arhgap25	Chrm4	Efs	Gfral	Igf2bp3	Mef2c	Olig2	Pxylp1	Slc2a4	Tmem260	
Arhgef1	Chrna3	Egr2	Gfy	Igfbp2	Mef2d	Olig3	Pygo1	Slc30a2	Tmem33	
Arhgef7	Chrna4	Egr3	Gins3	Igfbp5	Megf11	Onecut1	Rab11a	Slc30a3	Tmem53	
Arl5a	Chrna5	Egr4	Gja3	Igfbpl1	Megf8	Onecut3	Rab11fip4	Slc32a1	Tmem74b	
Arl8a	Chst1	Eif3f	Gjc1	Ikzf3	Meiob	Os9	Rab28	Slc35d3	Tmem81	
Arrb1	Chst11	Eif4e3	Gkap1	Il17ra	Meis1	Osbp2	Rab44	Slc35f3	Tmod1	
Art2b	Chst2	Elavl3	Gli1	Il25	Meis2	Osbpl6	Rab6b	Slc38a1	Tmod2	
Arx	Chst8	Elavl4	Gli2	Il6ra	Melk	Osr1	Ralgapa2	Slc38a3	Tmtc1	
Asb10	Cib4	Elfn1	Glipr2	Inha	Meox1	Ostm1	Rarres1	Slc41a2	Tnfaip8l1	
Asb18	Cilp2	Elfn2	Glis1	Inhbb	Mettl3	Otof	Rasal3	Slc43a1	Tnfrsf25	
Ascl5	Cited1	Elovl3	Glrx3	Insig2	Mfap5	Otop1	Rasd2	Slc45a2	Tnik	
Asic1	Ckb	Elovl4	Glyctk	Insm1	Mfsd4b1	Otop2	Rasgef1b	Slc47a1	Tnpo3	
Asic4	Clcn1	Emilin2	Gm10053	Insrr	Mfsd7c	Otp	Rasgef1c	Slc47a2	Tnrc6c	
Aspg	Cldn18	Emilin3	Gm10160	Ints3	Mgat5	Otub1	Rasgrp1	Slc52a3	Tns1	
Astl	Clec11a	Emx1	Gm10320	Iqcf6	Mgat5b	Otx1	Rax	Slc6a15	Tnxb	
Asxl3	Clec2l	Emx2	Gm10570	Irf4	Mgll	Otx2	Rbfox3	Slc6a20a	Tollip	
Atoh1	Clic4	En1	Gm10803	Irf5	Micall2	Oxtr	Rbm15	Slc6a20b	Tomm5	
Atp12a	Clstn1	En2	Gm11127	Irf8	Mkx	Pabpn1l	Rbm17	Slc6a4	Topaz1	
Atp2b2	Cmtr2	Engase	Gm11444	Irs4	Mlec	Pacsin1	Rbm20	Slc6a7	Tox	

Atp2b3	Cnih3	Enho	Gm11733	Irx3	Mmd	Palm2	Rcn1	Slc7a8	Tpbgl
Atp2b4	Cnot10	Enthd1	Gm12830	Irx4	Mmp17	Panx2	Rec114	Slc8a3	Tppp
Atp5g2	Cnpy1	Eomes	Gm15155	Isl2	Mmp24	Papd5	Reep1	Slc9a2	Tpsg1
Atp6v1b1	Cntfr	Epha4	Gm17190	Islr	Mmp9	Paqr9	Rem1	Slc9a9	Trabd
Atp6v1c2	Cntn2	Epha8	Gm17455	Islr2	Mnx1	Parp1	Ren1	Slco4a1	Trank1
Atp6v1f	Coch	Ephb2	Gm20696	Ism2	Morc2a	Parp11	Ret	Slco5a1	Tril
Atp8b3	Col12a1	Ephx4	Gm27027	Itga11	Morn5	Parp16	Rfx4	Slf2	Trim36
Atp9a	Col13a1	Epm2aip1	Gm35911	Itga9	Mpo	Pax1	Rfx6	Smad2	Trim37
Atp9b	Col14a1	Eras	Gm42517	Itpr2	Mpp6	Pax2	Rgl1	Smarca2	Trim54
Atxn7l2	Col15a1	Ercc6	Gm4353	Jak1	Mreg	Pax3	Rgma	Smarcad1	Trim67
Avp	Col20a1	Erg	Gm4881	Jazf1	Mroh4	Pax5	Rgs10	Smoc1	Trim71
Avpr1a	Col23a1	Erh	Gm5093	Jph4	Mroh9	Pax6	Rgs20	Smug1	Trmo
Axin2	Col27a1	Espn	Gm525	Kalrn	Mrps27	Pax7	Rgs6	Smyd2	Trnp1
B3galt1	Col2a1	Espnl	Gm6169	Kank4	Mrps9	Pbx1	Rgs9bp	Smyd3	Trp73
B3gat2	Col4a5	Esrrb	Gm7030	Kazald1	Msantd2	Pbx4	Rhbdl3	Snai2	Tspan33
B3gnt7	Col5a1	Etaa1	Gm9047	Kbtbd11	Msc	Pcdh19	Rhbg	Snx3	Tspear
B4galnt2	Col6a4	Etnk2	Gm9376	Kbtbd12	Msh2	Pcdhga3	Rhcg	Sobp	Tspoap1
Banp	Col8a2	Ets2	Gm9881	Kcna2	Msi1	Pced1b	Rhebl1	Soga1	Tsx
Barhl1	Colgalt2	Etv5	Gm9925	Kcnb1	Msi2	Pcolce2	Rhou	Sorcs2	Ttbk1
Barhl2	Commd10	Evx1	Gm9955	Kcnc3	Msr1	Pcsk1n	Rhpn1	Sost	Ttc17
Barx1	Comp	Exoc3l2	Gmppa	Kcnc4	Msra	Pdcd4	Ric8b	Sowahd	Ttc28
Barx2	Copz2	Exoc7	Gnal	Kcnd3	Msx1	Pde2a	Rimklb	Sox1	Ttc29
Batf	Cpeb3	Exosc2	Gnao1	Kcng1	Msx2	Pde4a	Rims1	Sox10	Ttc9b
Batf3	Cplx2	Exosc9	Gnas	Kcng3	Mt2	Pde4dip	Rims2	Sox12	Ttll6
Baz1b	Cpne5	Extl1	Gnat1	Kcnh2	Mtbp	Pdgfra	Rims4	Sox14	Tub
BC029722	Cpne6	Extl3	Gnl2	Kcnh3	Mturn	Pdp2	Rin3	Sox17	Tubb3
BC052040	Cpne7	Eya1	Golga7b	Kcnip3	Mvb12b	Pdx1	Rnf146	Sox18	Twist2
Bcan	Cpxm1	Faah	Gorasp2	Kcnj1	Myb	Pdzd2	Rnf149	Sox2	Txndc5
Bcas1	Cpz	Fabp3	Gosr2	Kcnj12	Myh13	Pdzd8	Rnf150	Sox6	Txnip
Bcat1	Crabp1	Fahd2a	Got1	Kcnj4	Myl2	Pdzk1ip1	Rnf152	Sox7	Ube2j1
Bcl11a	Cracr2a	Faim	Gp1bb	Kcnk1	Myof	Pecr	Rnf17	Sox8	Ube2ql1
Bcl6b	Creb3l1	Fam131b	Gpat3	Kcnk12	Myom2	Peli2	Rnf19b	Sox9	Ubl4b
Bend4	Creb5	Fam163a	Gpc5	Kcnk13	Nacc2	Penk	Rnf220	Sp2	Uchl1
Bhlhe22	Creg2	Fam163b	Gpr1	Kcnk15	Nags	Pfkfb3	Robo3	Sp6	Ucn
Bhlhe23	Crhr1	Fam169a	Gpr135	Kcnk18	Nanos2	Pgbd5	Robo4	Sp8	Ufsp1
Bhlhe41	Crhr2	Fam171b	Gpr155	Kcnk2	Nat8l	Pglyrp3	Ropn1	Sp9	Uhrf1bp1l
Bin3	Crispld2	Fam189a2	Gpr157	Kcnk3	Ncald	Phf12	Rora	Spaca7	Unc5a
Blk	Crlf1	Fam19a2	Gpr162	Kcnmb4	Ncan	Phf2	Rorb	Spag6	Uncx
Bloc1s3	Crtc1	Fam19a3	Gpr25	Kcnn1	Ncoa3	Phf21b	Ros1	Spag8	Upb1
Bmp10	Csgalnact1	Fam204a	Gpr50	Kcnn2	Ncs1	Phox2b	Rp1	Spata13	Urod
Bmp6	Csnk1e	Fam212b	Gpr84	Kcnq4	Ndrg4	Phpt1	Rpa1	Spata18	Ush1g
Bmp8a	Ctdspl	Fam43b	Gpr88	Kcp	Ndufa4l2	Phykpl	Rpap2	Spats2l	Usp46
Bnc1	Cth	Fam69c	Greb1	Kctd1	Ndufs6	Pianp	Rplp1	Spdef	Ust
Bnip1	Cux2	Fam78a	Grem1	Kctd12	Nefh	Pias1	Rragd	Specc1	Utf1
Bnip2	Cxcr4	Fam81a	Grin2c	Kctd15	Nek7	Pih1h3b	Rras2	Speg	Utp11
Boc	Cxxc4	Fam90a1b	Grin2d	Kdelr1	Nelfcd	Pik3cd	Rspo1	Spocd1	Utp6
Brd7	Cxxc5	Fam98b	Grm2	Kdm3a	Nes	Pin1	Rspo3	Spon1	Uvrag
Brf2	Cygb	Fars2	Grm4	Kdm5b	Neurl1a	Pitpnb	Rtbdn	Sppl2c	Vash1
Brip1	Cym	Fbln7	Grm6	Kdr	Neurl2	Pitx1	Rtn2	Spsb1	Vax1
Brsk2	Cyp24a1	Fbxl15	Grm8	Keap1	Neurod2	Pitx2	Rtn4r	Spsb4	Vax2
Bsn	Cyp26a1	Fbxl16	Grp	Kif13b	Neurog1	Pitx3	Rtn4rl1	Sptb	Veph1

Supplementary Table 6: GREAT analysis of H3K27me3 ChIP peaks, Negative Log(q value)

	MSigDB Signature Name	Genotype<br>ChIP	*Ezh2* WT<br>EZH2	*Ezh2* Het<br>EZH2	*Ezh2* WT<br>H3K27me3	*Ezh2* Het<br>H3K27me3	*Ezh2* Null<br>H3K27me3
DNA Damage Response, Cell Cycle and Stemness	FISCHER_DREAM_TARGETS		0.305	15.498	0.000	0.008	0.06511267
	PUJANA_BRCA1_PCC_NETWORK		3.137	12.085	0.000	0.053	0.00753809
	GRAESSMANN_APOPTOSIS_BY_DOXORUBICIN_UP		3.368	15.567	0.000	0.193	1.83863793
	PUJANA_ATM_PCC_NETWORK		3.324	16.086	0.045	0.246	0.1656196
	ENK_UV_RESPONSE_KERATINOCYTE_DN		2.130	15.396	0.101	0.452	0.06025526
	WEI_MYCN_TARGETS_WITH_E_BOX		1.926	15.816	0.139	0.592	0.43970006
	BENPORATH_CYCLING_GENES		2.435	11.655	0.228	0.385	0.24242402
	ZWANG_CLASS_1_TRANSIENTLY_INDUCED_BY_EGF		8.955	12.032	0.636	0.705	2.40074425
	BENPORATH_NANOG_TARGETS		2.300	16.738	1.397	0.284	0.34409114
	BUYTAERT_PHOTODYNAMIC_THERAPY_STRESS_UP		7.975	21.758	0.724	1.258	1.32692351
	NAGASHIMA_NRG1_SIGNALING_UP		1.477	14.696	0.067	1.662	0.8134012
	GRAESSMANN_APOPTOSIS_BY_DOXORUBICIN_DN		2.404	32.806	0.257	2.148	0.84023448
	KIM_WT1_TARGETS_UP		6.596	16.576	2.188	2.022	3.36089618
	HAMAI_APOPTOSIS_VIA_TRAIL_UP		1.123	14.463	2.243	1.815	0.09505622
	DACOSTA_UV_RESPONSE_VIA_ERCC3_COMMON_DN		11.750	23.253	4.466	4.770	2.00188085
	GOBERT_OLIGODENDROCYTE_DIFFERENTIATION_DN		9.382	20.722	2.580	10.194	2.7826927
	DACOSTA_UV_RESPONSE_VIA_ERCC3_DN		18.309	36.870	6.418	8.715	2.67154508
Canonical PRC2 Targets	MIKKELSEN_NPC_HCP_WITH_H3K27ME3		23.893	0.185	32.812	7.357	2.04401845
	BENPORATH_PRC2_TARGETS		29.245	0.003	50.527	19.736	1.46147904
	MEISSNER_NPC_HCP_WITH_H3K4ME2_AND_H3K27ME3		29.785	0.058	37.662	14.258	2.03322239
	BENPORATH_EED_TARGETS		31.873	0.021	58.927	26.296	1.9365808
	MIKKELSEN_MEF_HCP_WITH_H3K27ME3		32.348	0.011	70.938	20.176	1.67587038
	BENPORATH_SUZ12_TARGETS		32.452	0.039	78.863	29.115	3.7022519
	MIKKELSEN_MCV6_HCP_WITH_H3K27ME3		36.515	0.093	50.221	28.184	1.01935089
	BENPORATH_ES_WITH_H3K27ME3		39.326	0.036	78.153	34.530	2.37539656

Supplementary Table 7: Gene Set Enrichment Analysis for FOXP2 over-expressing cells

		HBEC3KT		BEAS2B		H460		H2030	
	MSigDB Signature Names	ES	negLog(q)	ES	negLog(q)	ES	negLog(q)	ES	negLog(q)
EMT + TGFβ	HALLMARK_EPITHELIAL_MESENCHYMAL_TRANSITION.grp	0.522	2.064	0.453	1.537	0.585	1.428	0.400	0.559
	GO_EPITHELIAL_TO_MESENCHYMAL_TRANSITION.grp	-0.251	0.318	0.468	1.374	0.518	0.838	0.291	0.215
	GO_POSITIVE_REGULATION_OF_MESENCHYMAL_CELL_ PROLIFERATION.grp	0.600	1.093	0.849	3.132	0.727	1.155	0.693	0.435
	ONDER_CDH1_TARGETS_2_UP.grp	0.474	1.608	-0.365	0.338	-0.401	0.287	-0.286	0.142
	CHARAFE_BREAST_CANCER_BASAL_VS_MESENCHYMAL_DN.grp	0.603	2.081	-0.371	0.354	0.589	0.930	0.307	0.153
	TAVAZOIE_METASTASIS.grp	0.505	1.345	0.470	0.984	0.754	2.357	-0.407	0.070
	WU_CELL_MIGRATION.grp	0.300	0.299	0.527	2.293	0.571	1.253	0.333	0.406
	BIOCARTA_TGFB_PATHWAY.grp	-0.360	0.235	-0.399	0.255	0.801	1.596	0.143	0.000
	KEGG_TGF_BETA_SIGNALING_PATHWAY.grp	-0.330	0.425	0.410	0.822	0.488	0.558	0.470	0.523
	TGFB_UP.V1_UP.grp	0.283	0.211	0.407	1.129	0.579	1.330	0.394	0.587
Lung Morphogenesis	GO_EPITHELIAL_CELL_PROLIFERATION_INVOLVED_IN_ LUNG_MORPHOGENESIS.grp	0.828	2.062	0.882	2.199	0.859	1.255	0.805	0.190
	GO_EPITHELIAL_TUBE_BRANCHING_INVOLVED_IN_LUNG_MORPH OGENESIS.grp	-0.354	0.354	0.632	1.436	0.610	0.630	-0.258	0.000
	GO_LUNG_ALVEOLUS_DEVELOPMENT.grp	0.533	1.161	0.638	1.982	0.576	0.711	0.495	0.568
	GO_LUNG_EPITHELIUM_DEVELOPMENT.grp	-0.370	0.320	0.791	3.125	0.503	0.427	0.439	0.376
MET + Cell Adhesion	ONDER_CDH1_TARGETS_2_DN.grp	0.345	0.680	0.625	4.000	0.685	4.000	0.353	0.544
	GO_POSITIVE_REGULATION_OF_EPITHELIAL_CELL_ PROLIFERATION.grp	0.251	0.108	0.542	2.251	0.632	1.632	0.417	0.553
	KEGG_ADHERENS_JUNCTION.grp	-0.218	0.124	0.232	0.036	0.625	1.274	-0.281	0.076
	KEGG_CELL_ADHESION_MOLECULES_CAMS.grp	0.635	2.781	0.519	1.408	0.534	0.739	-0.373	0.084
	GO_EXTRACELLULAR_MATRIX_STRUCTURAL_ CONSTITUENT.grp	0.693	4.000	0.419	0.986	0.452	0.508	-0.356	0.204
	KEGG_ECM_RECEPTOR_INTERACTION.grp	0.519	1.295	0.459	1.027	0.511	0.624	-0.286	0.056
TNFa + Inflamm.	HALLMARK_INFLAMMATORY_RESPONSE.grp	0.425	1.103	0.536	2.186	0.467	0.634	0.355	0.389
	HALLMARK_INTERFERON_GAMMA_RESPONSE.grp	0.460	1.435	0.343	0.726	0.373	0.316	-0.448	0.357
	HALLMARK_TGF_BETA_SIGNALING.grp	0.260	0.077	0.527	1.420	0.661	1.397	0.236	0.002
	HALLMARK_TNFA_SIGNALING_VIA_NFKB.grp	0.281	0.205	0.525	2.299	0.511	0.978	0.357	0.525
	PHONG_TNF_TARGETS_UP.grp	0.348	0.343	0.728	4.000	0.600	1.024	0.337	0.253
	TIAN_TNF_SIGNALING_VIA_NFKB.grp	0.590	1.268	0.815	3.309	0.773	1.557	0.404	0.265
	ZHANG_RESPONSE_TO_IKK_INHIBITOR_AND_TNF_UP.grp	0.359	0.672	0.577	3.229	0.469	0.716	0.416	0.513
Ras Signaling	HALLMARK_KRAS_SIGNALING_UP.grp	0.410	1.007	0.481	1.510	0.479	0.726	0.430	0.550
	KRAS.50_UP.V1_UP.grp	0.541	0.960	0.800	2.219	0.793	1.454	0.467	0.363
	KRAS.600.LUNG.BREAST_UP.V1_UP.grp	0.571	2.578	0.579	2.677	0.606	1.454	0.417	0.567
	KRAS.600_UP.V1_UP.grp	0.551	2.356	0.497	1.676	0.534	0.980	0.470	0.497
	KRAS.LUNG.BREAST_UP.V1_UP.grp	0.577	2.144	0.634	2.258	0.719	2.017	0.541	0.455
	KRAS.LUNG_UP.V1_UP.grp	0.602	2.374	0.528	1.478	0.679	1.372	0.507	0.568
	BILD_HRAS_ONCOGENIC_SIGNATURE.grp	-0.317	0.602	0.564	3.067	0.659	3.192	0.362	0.531

Supplementary Table 8: Primers used in this study

Genotyping Primers		
**Gene**	**Primer Name**	**Sequence**
Kras G12D	*Kras* 1	GTC TTT CCC CAG CAC AGT GC
	Kras 2	CTC TTG CCT ACG CCA CCA GCT C
	Kras 3	AGC TAG CCA CCA TGG CTT GAG TAA GTC TGC A
Ezh2 Floxed	*Ezh2* F	CCC ATG TTT AAG GGC ATA GTG ACA TG
	Ezh2 R	ATG TGC AGG TCA GTC AGC AAC TTC AG
	Ezh2 floxed excised	TCG AGG GAC CTA ATA ACT CGT ATA GC
P53 Floxed	*P53* A	CAC AAA AAC AGG TTA AAC CCA G
	P53 B	AGC ACA TAG GAG GCA GAG AC

Mouse ChIP Primers		
**Primer Name**	**Forward Primer Sequence**	**Reverse Primer Sequence**
Foxp2 5’ TSS	GCTGCTCTGCTCTCAGTGTG	CCACAACCCGGATCTCTGTA
Foxp2 Reg. Site	GCAAGTTGTAGGTAGGGTGC	ACAGCGACACAGATGGAATG
Foxp2 3’TSS	TGAACCTTTGTCACCCCTCA	GATCACGTCCCACACTGATG
Foxp2 ATG	CACTCCCCACAATGATGCTG	CTTGATCTCCCATCCCTGCT

Human ChIP Primers		
**Primer Name**	**Forward Primer Sequence**	**Reverse Primer Sequence**
FOXP2 Reg. Site	GGTCTCCCTTTCCGACTACC	AAAGCCGAGTTGTGTCTGCT
FOXP2 TSS	ACGGACGCCAAAACAATCAC	GCTCACTGTCAAAGCCACC
FOXP2 ATG	TGCTGTCTCTGTAATTGGCA	GCTGCTTAGAGTGCTCATTCC

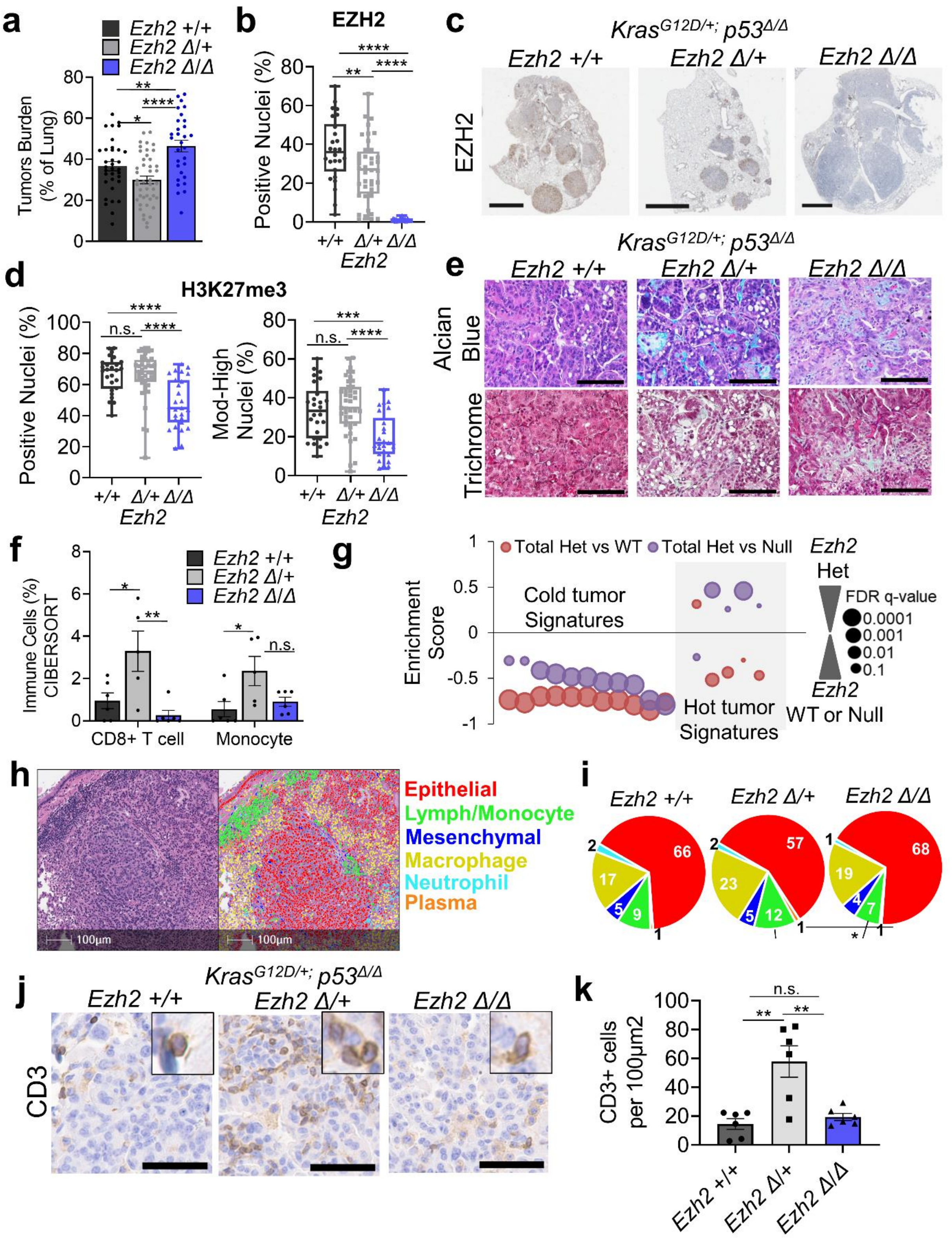

Supplementary Fig. 1: *Ezh2* Haplo- and Full-Insufficiency Drive Distinct Phenotypes in KRAS+/*Trp53*-null Lung Cancer

(a) Tumor burden was analyzed by ImageJ for indicated mice at days 83-133 post tumor induction and plotted as mean values +/- SEM. * indicates p=0.032 *Ezh2* heterozygous vs WT, ** indicates p=0.0098 *Ezh2* WT vs null, and **** indicates p=0.0000039 *Ezh2* heterozygous vs null by one-way ANOVA with multiple comparisons

and Holm-Šídák's *post-hoc* test. n=34, 44, 30 for *Ezh2* WT, heterozygous, null individual mice, respectively. **(b)** Average percentage of nuclei stained for EZH2 in mouse lung tumors with the indicated *Ezh2* genotypes plotted as box-and-whisker plots, error bars are min-max, box bounds are 25th and 75th percentiles and center line is median. ** indicates p=0.0032 *Ezh2* WT compared with heterozygous, **** indicates p=7E-15 for WT compared with *Ezh2* null, and p=9.35E-11 for heterozygous compared to *Ezh2* null by one-way ANOVA with multiple comparisons and Holm-Šídák's *post-hoc* test. n=26, 40, 27 for *Ezh2* WT, heterozygous, null individual mice, respectively. **(c)** Representative images of EZH2 immunostaining in mouse whole lung with the indicated genotypes. n are summarized in (b). Scale bar = 2mm. **(d)** Average percentage of positively (left) and moderately positively (right) stained tumor nuclei for H3K27me3 in mouse lung tumors with the indicated *Ezh2* genotypes. *** indicates p=0.0009; **** indicates p<0.00001 by one-way ANOVA with multiple comparisons and Holm-Šídák's *post-hoc* test. n=26, 41, 27 for *Ezh2* WT, heterozygous, null individual mice, respectively. **(e)** Representative images of alcian blue (mucins) and Masson's trichrome (connective tissue) stains in mouse tumor sections with the indicated genotypes, scale bar = 100µm. Stains were performed in n=7, 7, 6 for *Ezh2* WT, heterozygous, null individual mice, respectively. **(f)** Infiltrations of the indicated immune cell types were estimated by TIMER2.0 CIBERSORT analysis in the samples with indicated genotypes plotted as mean values +/- SEM. * indicates p=0.034 for monocytes, p=0.02 for CD8+, and ** indicates p=0.0056 by one-way ANOVA with multiple comparisons and Holm-Šídák's *post-hoc* test. n=6, 5, 6 for *Ezh2* WT, heterozygous, null individual mice, respectively. **(g)** GSEA plots evaluating the changes in the indicated "cold" or "hot" tumor gene signatures in total *Ezh2* heterozygous tumors compared with *Ezh2* WT and *Ezh2* null total tumors. Dot size reflect FDR estimates. Data are shown in Supplementary Table 1. **(h)** Representative HALO® image analysis of KRAS+/*Trp53*-null tumor (*Ezh2* +/+) shows the distribution of epithelial cells, mesenchymal cells, neutrophils, plasma cells, lymphoid cells or monocytes, and macrophages and **(i)** their percentages in the indicated genotype of *Ezh2*. * indicates p=0.01 by one-way ANOVA with multiple comparisons and Holm-Šídák's *post-hoc* test. n=8 individual mice per genotype, all between 116-132 days post adeno-Cre. **(j)** Representative images of CD3 staining, scale bar = 50µm. **(k)** Average counts of CD3+ cells per $100 µm^2$ in mouse tumors with the indicated genotypes plotted as mean values +/- SEM. ** indicates p=0.0023 by one-way ANOVA with multiple comparisons and Holm-Šídák's *post-hoc* test. n=6 individual mice per genotype, all between 116-132 days post adeno-Cre. Genotypes are signified as: WT +/+; heterozygous Δ/+; null, Δ/Δ. Source data are provided for this figure.

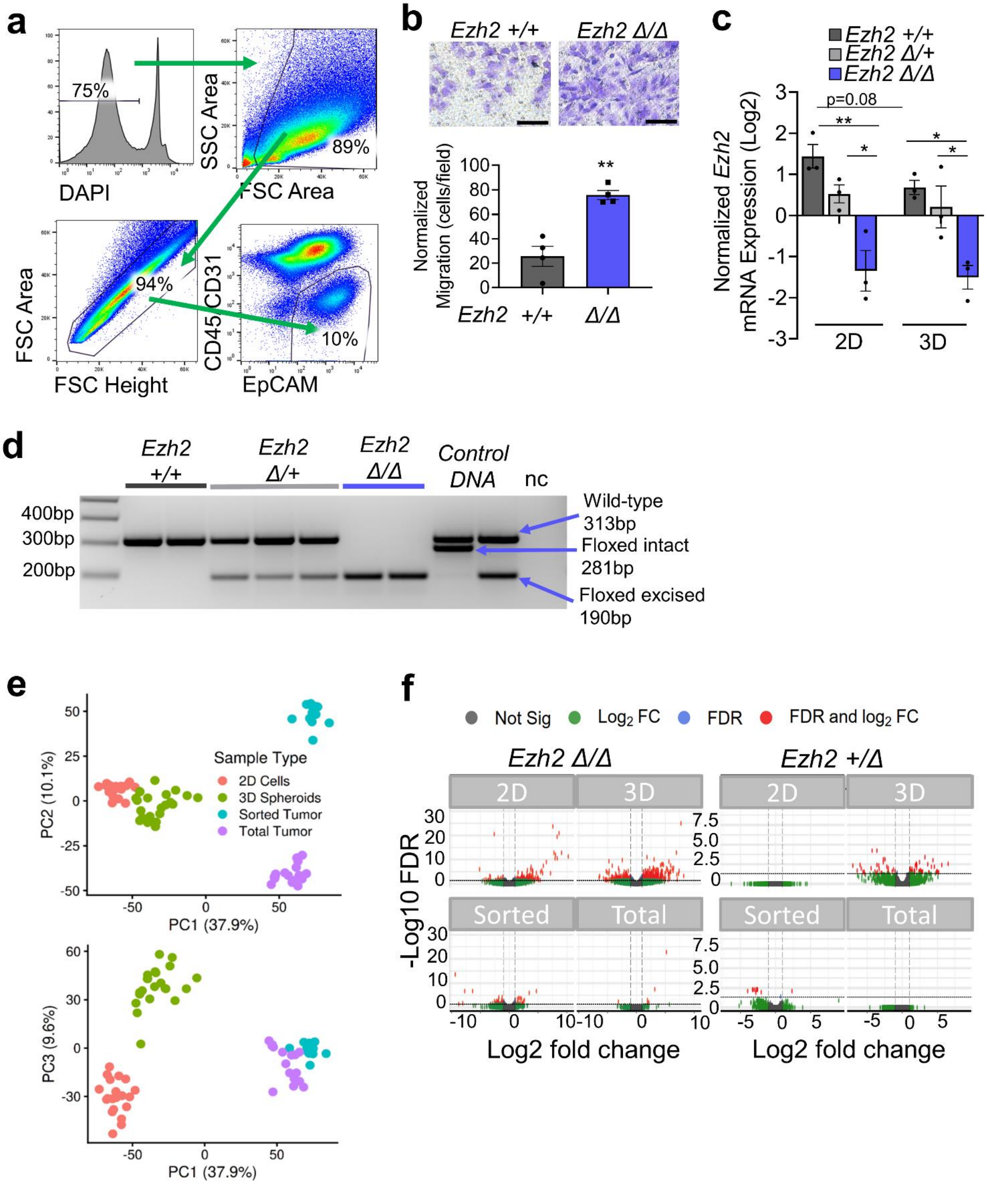

Supplementary Fig. 2: Model Systems Differ in Cell Cycle and Gene Expression

(a) Schematic for sorting of $EpCAM^{+}CD45^{-}CD31^{-}$ mouse primary tumor cells. **(b)** Representative images, scale bar = 100µm, and average numbers of migrated cells normalized to growth rates in *Ezh2* WT UK803 and *Ezh2* null UK777 mouse lines plotted as mean values +/- SEM. ** indicates p=0.0015 by two-tailed t-test. n=4 individual

cell cultures per group. **(c)** Expression of *Ezh2* mRNA in mouse primary 2D cells and 3D tumoroids of the indicated *Ezh2* genotypes graphed as mean values +/- SEM. * indicates $p<0.028$, ** indicates $p=0.0043$ by one-way ANOVA with multiple comparisons and Holm-Šídák's *post-hoc* test between genotypes and two-tailed t-test between conditions. n=3 individual mouse tumor-derived cultures for each genotype/culture system. **(d)** Genomic DNA PCR of WT, floxed intact and floxed excised *Ezh2* alleles in the tumoroids with the indicated genotype, and control DNA with known alleles. Results are representative of n=3 or greater individual mouse tumor-derived cultures for each genotype/condition. **(e)** Principal component (PC) analysis for the variability of gene expressions in the indicated model systems. Sample scores from PC1 and PC2 (left graph) and PC1 and PC3 (right graph) for a principal component decomposition of the log(TPM + 1) values for each sample are shown. n=17, 13, 20, 21 for total tumor, sorted tumor, 2D cell cultures, 3D tumoroid individual mouse tumor-derived samples, respectively. **(f)** Distributions of differentially expressed genes *Ezh2* null vs WT are shown as volcano plots in the indicated model systems. FDR cut-off is 0.05 and log2 fold change cut-off is 1. Genotypes are signified as: WT +/+; heterozygous Δ/+; null, Δ/Δ. Source data are provided for this figure.

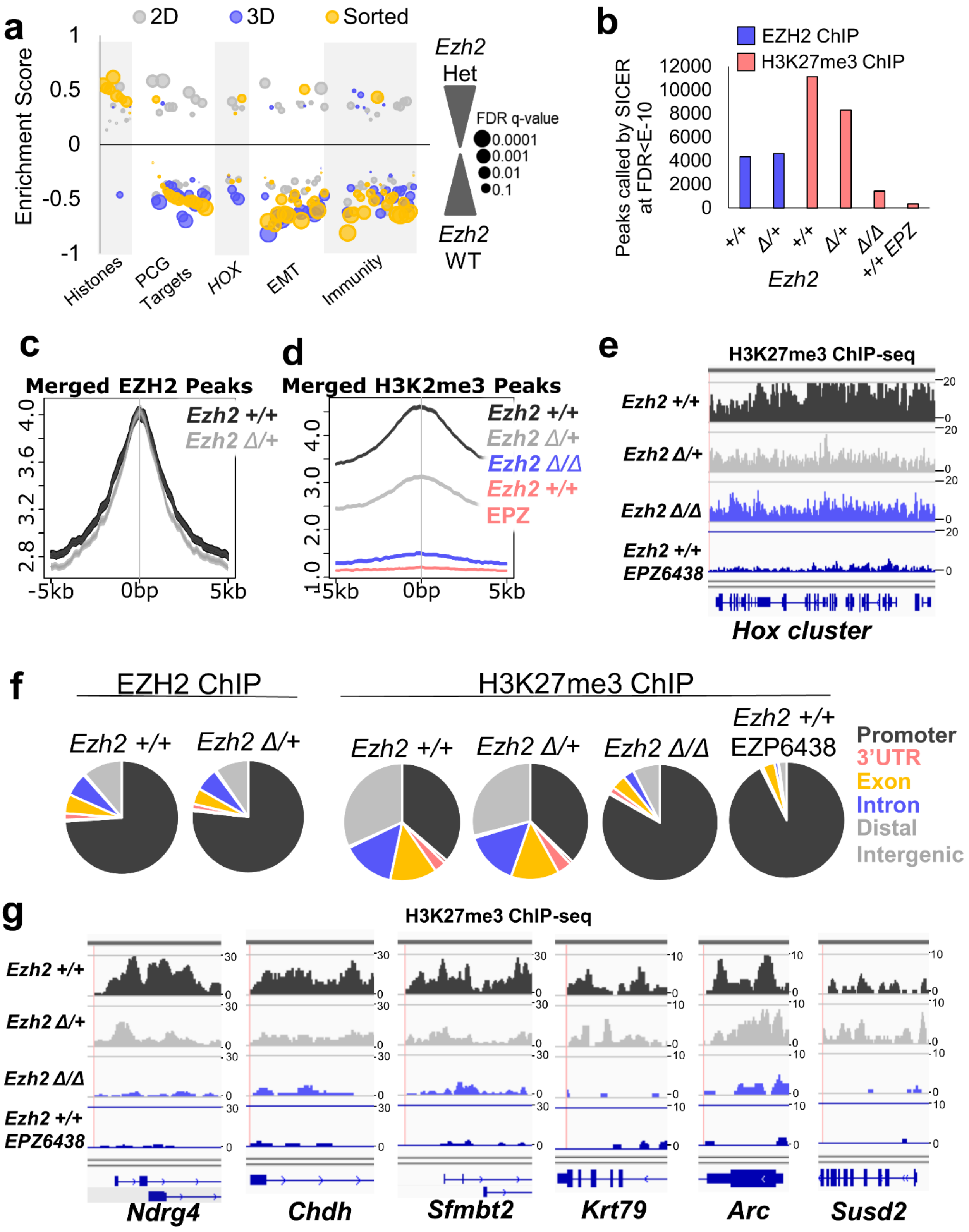

Supplementary Fig. 3: *Ezh2* Deficiency Leads to Diverse Gene Expression in Different Models

(a) Rank ordered gene lists were queried against the MSigDB databases and enrichment scores of selected gene signatures enriched in the *Ezh2* heterozygous vs *Ezh2* WT samples for the indicated model systems were plotted. Dot size estimates FDR. Data are shown in Supplementary Table 4. **(b)** EZH2 or H3K27me3 ChIP-seq

peaks called by SICER at FDR <E-10 in the indicated 3D tumoroid samples. **(c)** Merged EZH2 ChIP-seq peaks in the indicated 3D tumoroid samples. **(d)** Merged H3K27me3 ChIP-seq peaks in the indicated 3D tumoroid samples. **(e)** Visualization of H3K27me3 ChIP peaks over the *Hox* loci in the indicated tumoroid samples. **(f)** Proportions of EZH2 and H3K27me3 peaks distributed in the indicated regions for each *Ezh2* genotype. **(g)** Visualization of H3K27me3 ChIP-seq peaks enriched in *Ndrg4*, *Chdh*, *Sfmbt2, Krtl79, Arc,* and *Susd2* genes regions in the indicated 3D tumoroid samples. Genotypes are signified as: WT +/+; heterozygous Δ/+; null, Δ/Δ. Source data are provided for this figure.

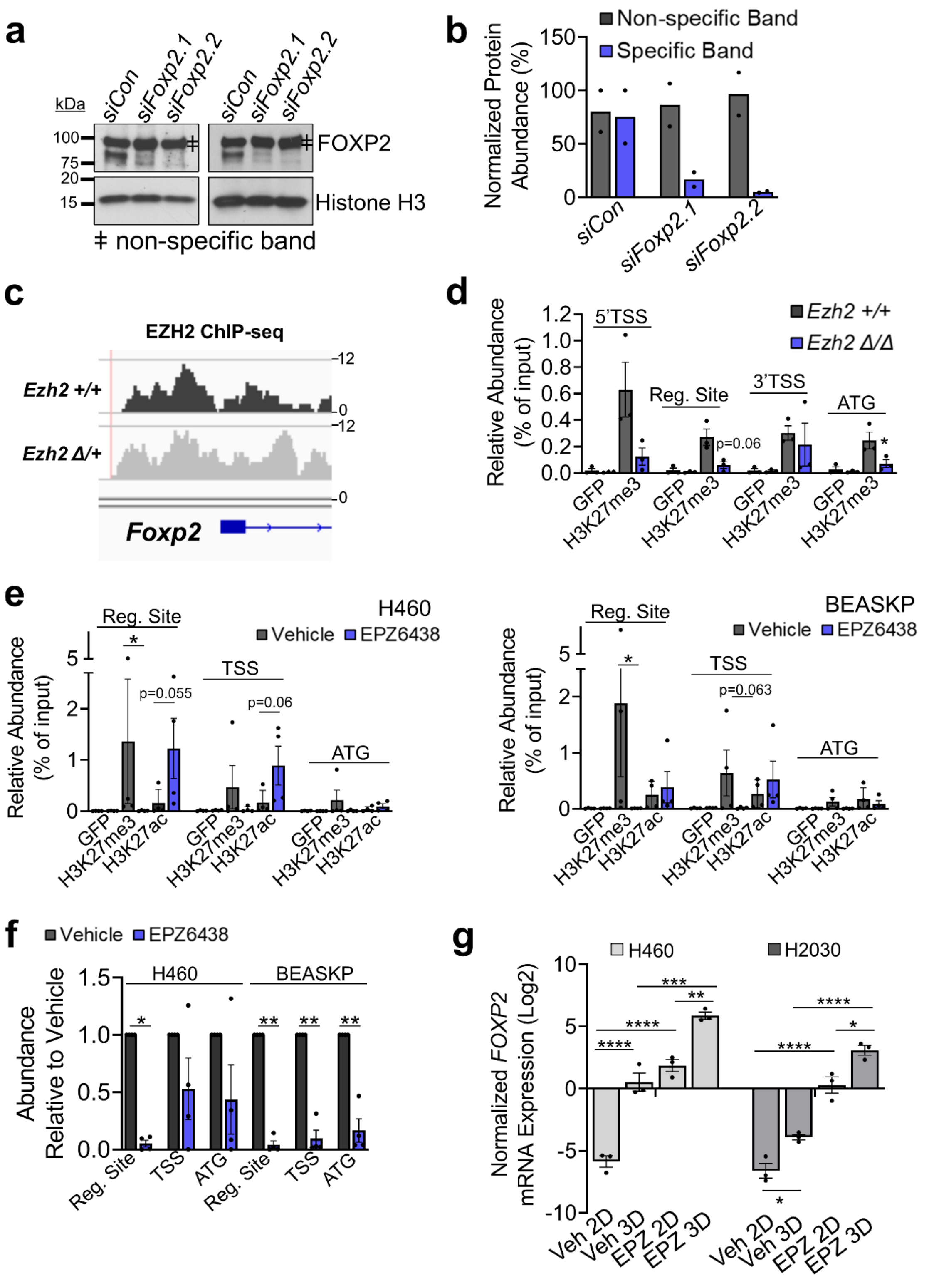

Supplementary Fig. 4: Loss of PRC2 Activity Drives Increased FOXP2 Expression in Normal and Malignant Lung Cells

(a) Immunoblot and **(b)** the normalized abundance of FOXP2 in the *Ezh2* null 2D mouse line UK777 treated with *siControl, siFoxp2.1* and *siFoxp2.2*. ‡ indicates the non-specific bands, n=2 blotting experiment from one individual cell culture per condition. **(c)** Visualization of ChIP-seq peaks for EZH2 occupancy at the FOXP2

transcription start site in *Ezh2* WT and *Ezh2* heterozygous tumoroids. A peak was called for EZH2 ChIP in the *Ezh2* WT sample at FDR=0.01. **(d)** ChIP-qPCR on 3D murine tumor spheroids with indicated *Ezh2* genotypes for *Foxp2* transcription start site (TSS) 5', regulatory element, TSS 3' and translation start site (ATG) using antibody against GFP and H3K27me3 plotted as mean values +/- SEM. * indicates p=0.049 *Ezh2* null vs *Ezh2* WT tumoroids by two-tailed t-test on log2-transformed values. n=3 individual mouse tumor-derived cultures per genotype. **(e)** ChIP-qPCR on human lines H460 (left) and BEASKP (right) with or without EPZ6438 treatment for *FOXP2* transcription start site (TSS) 5', regulatory element, TSS 3' and translation start site (ATG) using antibodies against H3K27ac, H3K27me3, and an IgG isotype control plotted as mean values +/- SEM. * indicates p=0.0138 EPZ6438 treatment vs vehicle by two-tailed t-test on log2 transformed values. n=4 individual cell cultures per group. **(f)** ChIP-qPCR on human lines from **(e)** normalized to the vehicle control sample. * indicates p=0.0112, ** indicates p<0.0071 by two-tailed t-test. **(g)** Normalized *FOXP2* mRNA expression of human lines H460 and H2030 grown in the indicated treatments and model systems, * indicates p<0.0196, ** p=0.0024, *** p=0.0004, ****p<0.0001 by one-way ANOVA with multiple comparisons and Holm-Šídák's *post-hoc* test. n=3 individual cell cultures per group. Genotypes are signified as: WT +/+; heterozygous Δ/+; null, Δ/Δ. Source data are provided for this figure.

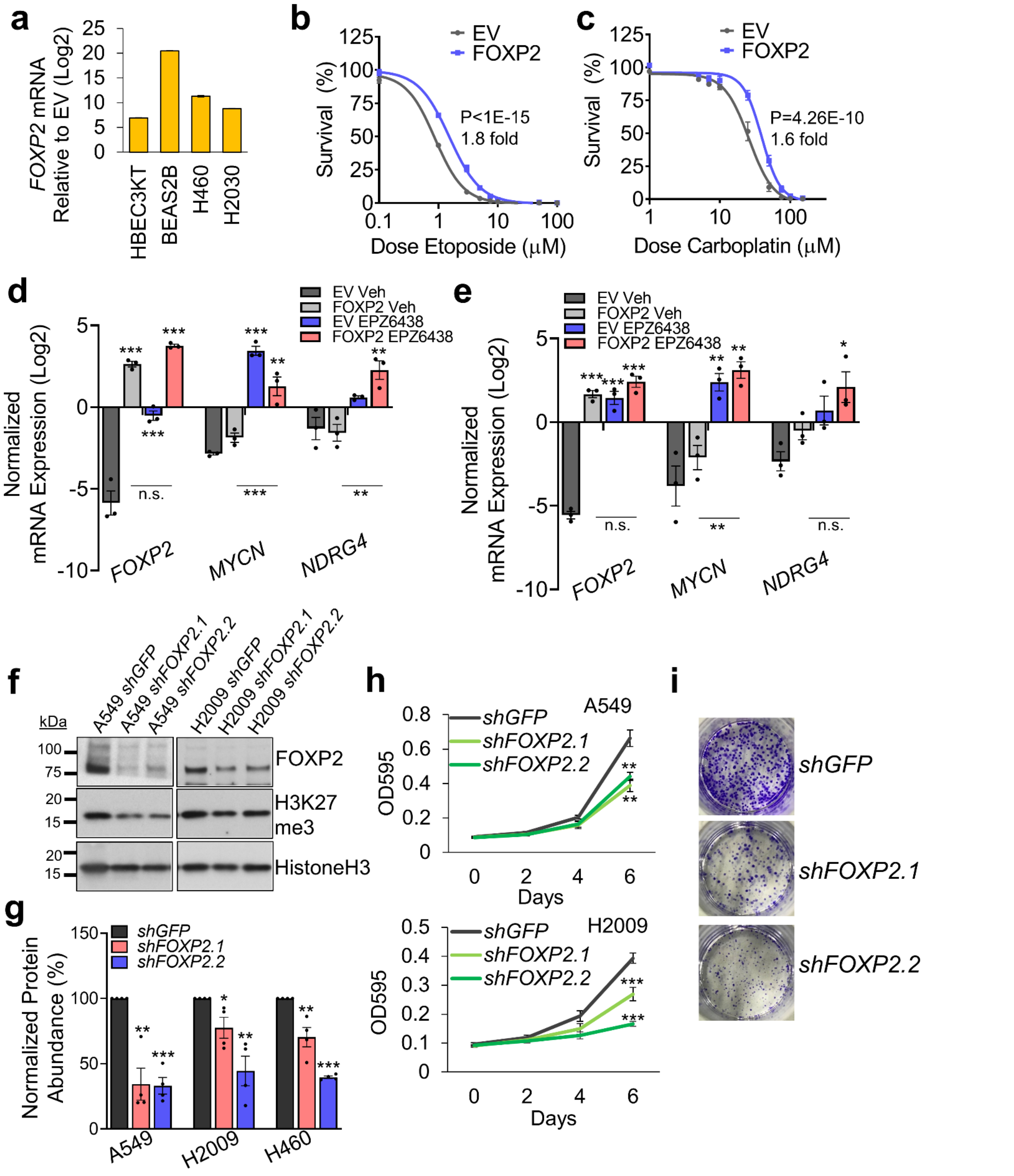

Supplementary Fig. 5: FOXP2 Drives Stemness and Migration in Lung Epithelial Cells

(a) Normalized *FOXP2* mRNA expression of FOXP2 over-expressed human lines compared to the empty vector, n=1 individual culture per cell line. **(b)** Dose-response to etoposide in BEAS2B cells with or without FOXP2

overexpression plotted as mean values +/- SEM. $p<1E-15$ compares IC_{50} values by extra sum-of-square F test. n=4 individual cultures per group. **(c)** Dose-response to carboplatin in BEAS2B cells with or without FOXP2 overexpression plotted as mean values +/- SEM. p=4.26E-10 compares IC_{50} values by extra sum-of-square F test. n=3 individual cell cultures per group. **(d and e)** Normalized mRNA expression of *FOXP2*, *NDRG4*, and *MYCN* with or without FOXP2 overexpression (oe) and with or without EPZ6438 treatment for **(d)** H460 and **(e)** H2030 3D samples graphed as mean values +/- SEM. * indicates p<0.02, ** indicates p<0.0062 and *** indicates p<0.0001 by one-way ANOVA with multiple comparisons and Holm-Šídák's *post-hoc* test. n=3 individual cell cultures per group. **(f)** Immunoblot and **(g)** normalized protein abundance in the indicated human lines transduced with small hairpins plotted as mean values +/- SEM. *shGFP* is the control cell line. * indicates p=0.03, ** p<0.008, *** p<0.0001 by two-tailed t-test. n=4 blotting experiments per cell line from one set of cell cultures for H460 and three sets of cell cultures for H2009 and A549. **(h)** Crystal violet growth assays were performed on 2D A549 and H2009 cells with or without FOXP2 knockdown at indicated days of culture plotted as mean values +/- SEM. ** indicates p=0.0021 *shGFP vs shFOXP2.1* and p=0.0099 *shGFP vs shFOXP2.2*, *** indicates p=0.00066 *shGFP vs shFOXP2.1* and p=0.00022 *shGFP vs shFOXP2.2* by two tailed t-test. n=4 individual cell cultures. **(i)** Representative whole well (standard 12-well plate) images of crystal violet stained H460 cells with indicated small hairpins 6 days after seeding. Source data are provided for this figure.

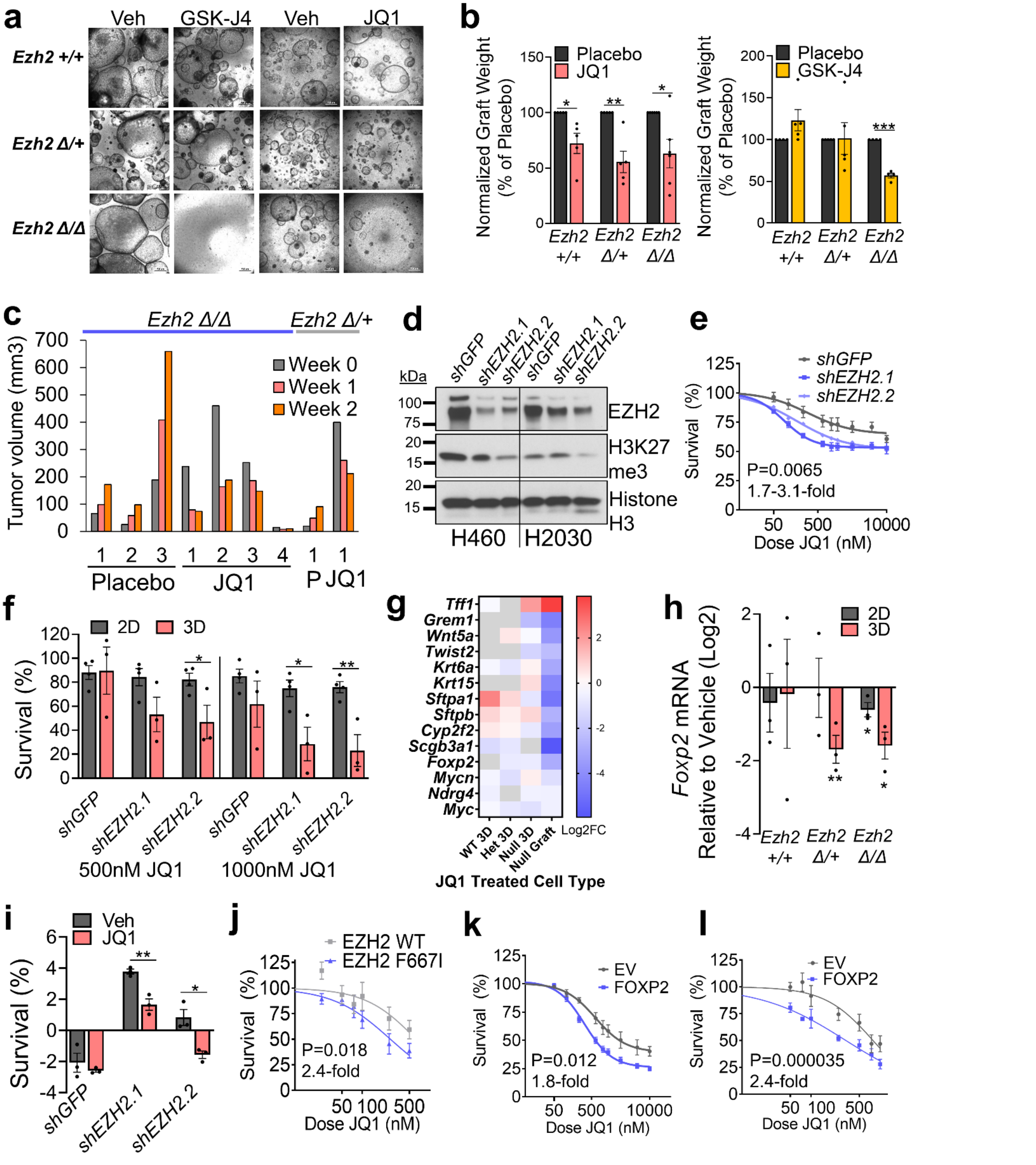

Supplementary Fig. 6: Polycomb Deficiency Drives BET and JMJD3/UTX Inhibitor Sensitivity

(a) Images of placebo-, GSK-J4- and JQ1-treated spheroids of the indicated *Ezh2* genotypes, scale bar=100µm.

(b) Normalized wet weights of placebo-, GSK-J4- and JQ1-treated *in vivo* grafts of the indicated *Ezh2* genotypes

plotted as mean values +/- SEM. * indicates p=0.0162 for Ezh2 WT p=0.0166 for *Ezh2* null, ** indicates p=0.001769, and *** indicates p=0.0000056 by two-tailed t-test. GSK-J4: n=4, 5, 4; JQ1: n=5, 5, 6 for *Ezh2* WT, heterozygous, null individual mouse tumor-derived grafts, respectively. **(c)** Quantification of tumor size in mouse lungs based on MRI at 0, 1 and 2 weeks of the indicated treatments. **(d)** H2030 and H460 2D cell lines with or without EZH2 knock-down using two different short-hairpins were examined for the expression of the indicated proteins by immunoblot. *shGFP* is the control cell line. Data representative of two independent cell cultures per group. **(e)** Dose-response to JQ1 in H2030 cells with or without EZH2 knock-down. IC_{50} values from 3 individual cell cultures were compared, p=0.0065 by extra sum-of-square F test. **(f)** Relative cell survival of H460 cells with or without EZH2 knock-down in 3D (n=3 individual cell cultures) and 2D (n=4 individual cell cultures) comparing JQ1 treatment compared to vehicle control with mean values +/- SEM plotted. * indicates p<0.045 and * indicates p=0.00772 by two-tailed t-test. **(g)** Heatmap depicting log-fold change (LFC) of significantly differentially expressed genes after JQ1 treatment in the indicated genotypes and models relative to control. n=1 matched pair of JQ1 treated and vehicle treated for each column. **(h)** Expression of *Foxp2* mRNA in mouse 2D cells and 3D tumoroids for the indicated *Ezh2* genotypes treated with vehicle or 100nM JQ1. * indicates p<0.034, ** indicates p=0.001 with two-tailed t-test. n=3 individual mouse tumor-derived cultures per group. **(i)** Expression of *FOXP2* mRNA in 3D H2030 cells with or without *EZH2* knock-down, and treated with vehicle or 100nM JQ1. * indicates p=0.013, ** indicates p=0.006 by two-tailed t-test. n=3 individual cell cultures per group. **(j)** Dose-response to JQ1 of 3D *Ezh2* null tumoroids rescued with EZH2 WT or EZH2 methyltransferase dead (F677I) plotted as mean values +/- SEM. p=0.018 comparing IC_{50} values by extra sum-of-square F test. n=4 individual cell cultures per group. **(k)** Dose-response to JQ1 of 2D H460 cells with or without FOXP2 overexpression plotted as mean values +/- SEM. p=0.012 comparing IC_{50} values by extra sum-of-square F test. n=4 individual cell cultures per group. **(l)** Dose-response to JQ1 of 3D H460 tumoroids with or without FOXP2 overexpression plotted as mean values +/- SEM. p=0.000035 comparing IC_{50} values by extra sum-of-square F test. n=4 individual cell cultures per group. Genotypes are signified as: WT +/+; heterozygous Δ/+; null, Δ/Δ. Source data are provided for this figure.

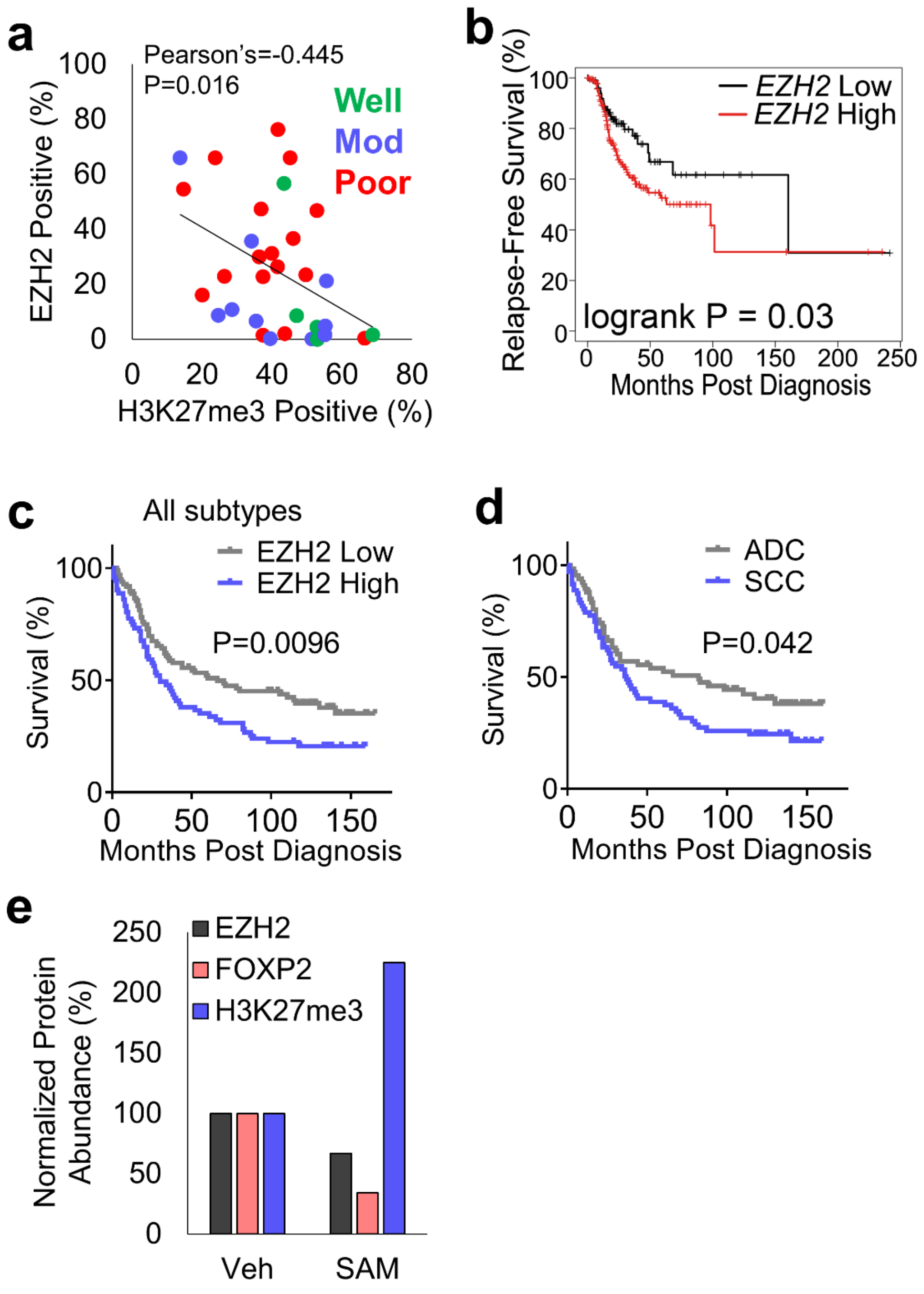

Supplementary Fig. 7: Decoupling of EZH2 from PRC2 Activity May Explain Co-Expression of EZH2 and FOXP2 in Patient Samples

(a) In tumors that were FOXP2 positive (>10% of nuclei), the correlation between EZH2 and H3K27me3 is shown. n=49 individual tumors. **(b)** Kaplan-Meier relapse-free survival curves for the *EZH2*-high and *EZH2*-low lung adenocarcinoma tumors as measured by RNA-sequencing 250 months post diagnosis. Groups were split at best cut-off, p value was calculated by log-rank test. n=366 individual tumors. **(c)** Kaplan-Meier lung cancer-specific overall survival curves for EZH2-high and EZH2-low tumors as measured by IHC; positivity was defined as at least 20% EZH2 positive tumor nuclei. The p value was calculated by log-rank test. n=167 individual tumors.

(d) Kaplan-Meier lung cancer-specific overall survival curves for ADC and SCC tumor groups in the TMA, p value was calculated by log-rank test. n=137 individual tumors. **(e)** The normalized protein abundance of the indicated protein expression in 2D *Ezh2* WT UK803 cells treated with or without 500μM SAM treatment for 6 days. Source data are provided for this figure.
