# Supplementary material for: Polycomb deficiency drives a FOXP2-high aggressive state targetable by epigenetic inhibitors
Source: Nat Commun. 2023 Jan 20;14:336. doi: 10.1038/s41467-023-35784-x (PMC9859827; doi:10.1038/s41467-023-35784-x)
Supplement: Supplementary file 3 — Reporting Summary [file 41467_2023_35784_MOESM3_ESM.pdf]

nature research

Corresponding author(s): Christine Fillmore Brainson

Last updated by author(s): Dec 17, 2022

Reporting Summary

Nature Research wishes to improve the reproducibility of the work that we publish. This form provides structure for consistency and transparency in reporting. For further information on Nature Research policies, see our Editorial Policies and the Editorial Policy Checklist.

Statistics

For all statistical analyses, confirm that the following items are present in the figure legend, table legend, main text, or Methods section.

n/a	Confirmed	
☐	☒	The exact sample size (n) for each experimental group/condition, given as a discrete number and unit of measurement
☐	☒	A statement on whether measurements were taken from distinct samples or whether the same sample was measured repeatedly
☐	☒	The statistical test(s) used AND whether they are one- or two-sided<br>*Only common tests should be described solely by name; describe more complex techniques in the Methods section.*
☐	☒	A description of all covariates tested
☐	☒	A description of any assumptions or corrections, such as tests of normality and adjustment for multiple comparisons
☐	☒	A full description of the statistical parameters including central tendency (e.g. means) or other basic estimates (e.g. regression coefficient) AND variation (e.g. standard deviation) or associated estimates of uncertainty (e.g. confidence intervals)
☐	☒	For null hypothesis testing, the test statistic (e.g. F, t, r) with confidence intervals, effect sizes, degrees of freedom and P value noted<br>*Give P values as exact values whenever suitable.*
☒	☐	For Bayesian analysis, information on the choice of priors and Markov chain Monte Carlo settings
☒	☐	For hierarchical and complex designs, identification of the appropriate level for tests and full reporting of outcomes
☐	☒	Estimates of effect sizes (e.g. Cohen's d, Pearson's r), indicating how they were calculated

Our web collection on statistics for biologists contains articles on many of the points above.

Software and code

Policy information about availability of computer code

Data collection	Sequencing reads were trimmed and filtered using Trimmomatic (V0.39) to remove adapters and low quality reads.
Data analysis	Reads from human and mouse samples were mapped to Ensembl GRCh38 and GRCm38 transcripts annotation (release 82), respectively, using RSEM (1.3.2). Gene expression data normalization and differential expression analysis were performed using the R (3.6.3) package edgeR (3.28.1). Significantly up/downregulated genes were determined as log-fold change >= 0.5 and q-value < 0.05.<br><br>Principal components analysis and correlation analysis were performed using R (v 4.0.0), and functions available in visualizationQualityControl (v 0.3.6, https://github.com/moseleybioinformaticslab/visualizationQualityControl). Gene ontology enrichments used categoryCompare2 (commit 906721, https://github.com/moseleybioinformaticslab/categoryCompare2), as well as Bioconductor (v 3.11), org.Mm.eg.db (v 3.11.4), and GO.db (v 3.11.4). Other R packages used directly include: rmarkdown (v 2.1); dplyr (v 1.0.10); viridis (v 0.6.2); circlize (v 0.4.15); ComplexHeatmap (v 2.4.3); readr (v 2.1.3); ggplot2 (v 3.4.0); dendsort (v 0.3.3). All direct code, as well as inputs and outputs necessary for the principal components analysis are available from a figshare repository at https://doi.org/10.6084/m9.figshare.13179989.v1.

For manuscripts utilizing custom algorithms or software that are central to the research but not yet described in published literature, software must be made available to editors and reviewers. We strongly encourage code deposition in a community repository (e.g. GitHub). See the Nature Research guidelines for submitting code & software for further information.

Data

Policy information about availability of data

All manuscripts must include a data availability statement. This statement should provide the following information, where applicable:

- Accession codes, unique identifiers, or web links for publicly available datasets
- A list of figures that have associated raw data
- A description of any restrictions on data availability

The RNA-sequencing and ChIP-sequencing generated during this study are available at NCBI GEO database under accession numbers: GSE154689 (token ufefqwsaxdypxml) and GSEGSE182819 (token mlgtwkiaxzifxun). Five additional sorted tumor samples were excluded from the dataset because the sort operator that day was different and the samples did not cluster with all other total and sorted tumor samples. Those data are available upon request.

GSEA was performed with GSEA version 4.0.3 (Broad Institute) with rank-ordered gene lists generated using all log-fold change values. Mouse_ENSEMBL_Gene_ID_to_Human_Orthologs_MSIGDB.v7.1.chip was used to map mouse genes to human orthologs. Databases queried included Hallmarks (h.all.v7.1), Curated (c2.all.v7.1), BioCarta (c2.cp.biocarta.v7.1), KEGG (c2.cp.kegg.v7.1), Reactome (c2.cp.reactome.v7.1), GO (c5.all.v7.1), Oncogenic Signatures (c6.all.v7.1), and Immunologic Signatures (c7.all.v7.1). MSigDB: https://www.gsea-msigdb.org/gsea/msigdb/.

Field-specific reporting

Please select the one below that is the best fit for your research. If you are not sure, read the appropriate sections before making your selection.

- [x] Life sciences
- [ ] Behavioural & social sciences
- [ ] Ecological, evolutionary & environmental sciences

For a reference copy of the document with all sections, see nature.com/documents/nr-reporting-summary-flat.pdf

Life sciences study design

All studies must disclose on these points even when the disclosure is negative.

Sample size

No statistical methods were used to predetermine sample size. A sample size of 3-6 replicates was used for the majority of experiments as this sample size has allowed for measurement of statistically significant differences in similar previous studies such as PMCID: PMC8596110; PMC5385585; PMC4393352; PMC8743034.

Data exclusions

Data exclusions and reasoning are as follows:
RNA-sequencing data of five sorted samples of varied genotypes were excluded due to questions about sorter operation that day and data are available upon request. One Ezh2 null tumoroid that was an outlier in both in vitro and in vivo drug treatment experiments was excluded from genotype comparison analyses, and for one tumoroid line that was sequenced very early in development and later, only the later sample was kept in the RNA-seq genotype comparison. These FASTQ files are uploaded to GEO.
One replicate of A549 and H2009 shFOXP2 crystal violet growth was excluded due to addition of more media to the wells changing the overall readings but not the relative growth to the control line.
Two grafts tumors were recorded but had not met the starting volume of 10mm3 prior to treatment initiation and were therefore excluded from final analysis. They were both GSK-J4 vehicle control tumors.
Two runs of murine Ezh2-null cells grown in 3D and treated with JQ1 were excluded due to inefficacy of the drug (>70% alive at highest drug dose).

Replication

Experimental replicates are all explicitly stated in the figure legends. All replicates were successful with the exceptions noted above. Experimental replicates are all explicitly stated in the figure legends. For mouse experiments, the biological n represents individual donor mice, and often several replicate experiments for each donor mouse were averaged to produce the biological replicates. Each experiment was performed in at least experimental duplicate (i.e. individual cell cultures or RNAi sequences), biological duplicate (i.e. individual mouse or human donor) or both.

Randomization

Cell samples were allocated randomly into treatment groups. For subcutaneous tumor growth, baseline tumors were measured and mice were randomized into groups having relatively equal starting tumor burden. For MRI, mice for each cohort were measured at baseline and assigned into treatment groups so that starting tumor burden was roughly equivalent in the groups, with a preference for higher tumor burden assigned to experimental treatment. This was done to prevent high tumor burden mice from reaching endpoint before the experiment was complete (as they may have on placebo treatment) and to test if the drugs could work on larger tumors, which may be more challenging than shrinking smaller tumors.

Blinding

Blinding for murine immunostain imaging was not possible because the person most qualified to identify the tumor areas also knew the mouse numbers and tissue appearance. Blinding was used for the tissue microarray imaging. For both mouse and human studies, computer based stain quantification was used to measure relative stains in unbiased ways. Blinding was not used for animal experiments because the same person measured tumors and administered drugs.

Reporting for specific materials, systems and methods

We require information from authors about some types of materials, experimental systems and methods used in many studies. Here, indicate whether each material, system or method listed is relevant to your study. If you are not sure if a list item applies to your research, read the appropriate section before selecting a response.

Materials & experimental systems

n/a	Involved in the study
	[x] Antibodies
	[x] Eukaryotic cell lines
[x]	Palaeontology and archaeology
	[x] Animals and other organisms
	[x] Human research participants
[x]	Clinical data
[x]	Dual use research of concern

Methods

n/a	Involved in the study
	[x] ChIP-seq
	[x] Flow cytometry
[x]	MRI-based neuroimaging

Antibodies

Antibodies used

Anti-FOXP2 for human (Sigma Aldrich Cat# HPA000382; RRID:AB_1078908);
Anti-FOXP2 mouse and human (Cell Signaling Technology Cat# Cat# 5337, RRID:AB_10706940);
Anti-EZH2 (Cell Signaling Technology Cat# 5246S; RRID:AB_10694683);
Anti-H3K27me3 (Cell Signaling Technology Cat# 9733S; RRID:AB_2616029),
Anti-H3K27me3 (Millipore Cat# 07-449; RRID:AB_310624);
Anti-H3K27ac (AbCam Cat# ab4729; RRID:AB_2118291);
Anti-PCNA (Biolegend Cat# 307901; RRID:AB_314691);
Anti-Phospho Histone H3 (GeneTex Cat# GTX128116; RRID:AB_2885709);
Anti-Histone H3 (AbCam Cat# ab1791; RRID:AB_302613);
Anti-EZH2 (Active Motif Cat#39901; RRID:AB_2614956);
Anti-H3K27me3 (Active Motif Cat#39155; RRID:AB_2561020);
Anti-GFP (Thermo-Fisher #MS-1288-P0; RRID:AB_63267);
Anti-Rabbit IgG, HRP-linked (Novus Cat# NB7160; RRID:AB_10124655);
APC anti-mouse CD31 (Biolegend Cat# 102510; RRID:AB_312917);
APC anti-mouse CD45 (Biolegend Cat# 103112; RRID:AB_312977);
PE/Cy7 anti-mouse CD326/Ep-CAM (Biolegend Cat# 118216; RRID:AB_1236471).

Validation

Antibodies used in this study have been validated by size of band on Western Blot (FOXP2, EZH2, H3K27me3, H3K27ac, Histone H3, SUZ12, EED) and by knock-out or knock-down experiments (FOXP2, EZH2, H3K27me3), and by cellular localization on immunostainning (EZH2, H3K27me3, FOXP2) all shown in this paper. The human FOXP2 antibody is used as an established Human Protein Database anti; body. The flow cytometry antibodies have been used extensively in the literature and are known to mark the specific cell types of the lung (CD45, leukocyte, EpCAM, epithelial cells, CD31, endothelial cells). Please see other details below.

Anti-FOXP2 for human (Sigma Aldrich Cat# HPA000382; RRID:AB_1078908) Indicated Applications: Immunohistochemistry, Western Blot https://www.sigmaaldrich.com/US/en/product/sigma/hpa000382 "Every Prestige Antibody is tested in the following ways:
1) IHC tissue array of 44 normal human tissues and 20 of the most common cancer type tissues 2) Protein array of 364 human recombinant protein fragments."
Validated by Human Protein Atlas for human IHC, and by this manuscript for mouse IHC by localization to nuclei and tracking with RNA-expression by immunostaining. This manuscript also validated for western blotting by over-expression and knock-down studies that showed a band at the correct molecular weight.

Anti-FOXP2 mouse and human (Cell Signaling Technology Cat# 5337, RRID:AB_10706940) Indicated Applications: Western Blot, Immunoprecipiation https://www.cellsignal.com/products/primary-antibodies/foxp2-d55h9-rabbit-mab/5337 "FoxP2 (D55H9) Rabbit mAb detects endogenous levels of total FoxP2 protein."
In this manuscript we show that this antibody produces a specific and non-specific band for FOXP2 using human and mouse lysates for western blotting. PMCID: PMC9531268

Anti-EZH2 (Cell Signaling Technology Cat# 5246S; RRID:AB_10694683) Indicated Applications: Western Blot, Immunoprecipitation, Immunohistochemistry, Immunofluorescence, Flow Cytometry, Chromatin Immunoprecipitation, Chromatin Immunoprecipitation Seq, Cut and Run https://www.cellsignal.com/products/primary-antibodies/ezh2-d2c9-xp-rabbit-mab/5246 "Ezh2 (D2C9) XP® Rabbit mAb detects endogenous levels of total Ezh2 protein. This antibody does not cross-react with Ezh1 protein."
We had previously shown specificity, and show in this manuscript specificity for EZH2 by knock-down and knock-out studies in mouse and human cells in western blotting and IHC applications. PMCID: PMC5385585; PMC4393352

Anti-H3K27me3 (Cell Signaling Technology Cat# 9733S; RRID:AB_2616029) Indicated Applications: Western Blot, Immunohistochemsitry-Leica Bond, Immunohistochemistry, Immunofluorescence, Flow Cytometry, Chromatin IPleasemmmunoprecipitation, Chromatin Immunoprecipitation-seq, Cut and Run https://www.cellsignal.com/products/primary-antibodies/tri-methyl-histone-h3-lys27-c36b11-rabbit-mab/9733 "Tri-Methyl-Histone H3 (Lys27) (C36B11) Rabbit mAb (Alexa Fluor® 647 Conjugate) recognizes endogenous levels of histone H3 only when tri-methylated at Lys27. The antibody does not cross-react with non-methylated, mono-methylated or di-methylated Lys27. In addition, the antibody does not cross-react with mono-methylated, di-methylated or tri-methylated histone H3 at Lys4, Lys9, Lys36 or histone H4 at Lys20."
We had previously shown specificity, and show in this manuscript specificity for H3K27me3 by EZH2 knock-down, knock-out and

inhibition studies in mouse and human cells in western blotting and IHC applications. PMCID: PMC5385585; PMC8743034

Anti-H3K27me3 (Millipore Cat# 07-449; RRID:AB_310624) Indicated Applications: Immunocytochemistry, Immunohistochemsitry, Immunoprecipiation, Multiplexing, Western Blot https://www.sigmaaldrich.com/US/en/product/mm/07449 "Anti-trimethyl-Histone H3 (Lys27), also known as Anti-H3K27me3, is a highly published Rabbit Polyclonal Antibody. This protein A purified antibody is dot blot tested for trimethylated lysine 27 specificity and validated in WB, ICC, IP."
We had previously shown specificity for H3K27me3 by EZH2 knock-down and inhibition studies in mouse and human cells in western blotting applications. PMCID: PMC4393352; PMC5385585

Anti-H3K27ac (AbCam Cat# ab4729; RRID:AB_2118291) Indicated Applications: Immunocytochemistry, Immunofluorescence, Western Blot, Chromatin Immunoprecipitation, Immunohistochemistry https://www.abcam.com/histone-h3-acetyl-k27-antibody-chip-grade-ab4729.html "Reacts with: Mouse, Human; Application: ChIP" also called "ChIP Grade".
See PMCID: PMC5385585 for previous ChIP with this antibody.

Anti-PCNA (Biolegend Cat# 307901; RRID:AB_314691) Indicated Applications: Immunocytochemistry, Immunohistochemistry, Immunoprecipitation, Western Blot https://www.biolegend.com/nl-nl/explore-new-products/purified-anti-human-mouse-rat-pcna-antibody-816 "Verified Reactivity: Human, Mouse, Rat; IP, WB, IHC - Reported in the literature, not verified in house"
See PMCID: PMC6453568 for western blotting application and this manuscript showing that expression tracked with cell cycle changes.

Anti-Phospho Histone H3 (GeneTex Cat# GTX128116; RRID:AB_2885709) Indicated Applications: Western Blot, Immunocytochemistry, Immunohistochemsitry, Immunoprecipitation, Chromatin Immunoprecipitation https://www.genetex.com/Product/Detail/Histone-H3S10ph-phospho-Ser10-antibody/GTX128116 "Reactivity: Human, Mouse; Application: WB"
See PMID: 25323962 for western blotting application and this manuscript showing that expression tracked with cell cycle changes.

Anti-Histone H3 (AbCam Cat# ab1791; RRID:AB_302613) Indicated Applications: Immunocytochemistry, Immunohistochemsitry, Chromatin Immunoprecipitation, Immunoprecipitation, Western Blot https://www.abcam.com/histone-h3-antibody-nuclear-marker-and-chip-grade-ab1791.html "Reacts with: Mouse, Rat, Human....Detects a band of approximately 17 kDa (predicted molecular weight: 15 kDa).Can be blocked with Human Histone H3 peptide (ab12149)."
This is a widely used H3 antibody that is suitable for western blotting. PMCID: PMC5385585; PMC4393352; PMC8743034

Anti-EZH2 (Active Motif Cat#39901; RRID:AB_2614956) Indicated Applications: Immunohistochemsitry, Chromatin Immunoprecipitation, Chromatin Immunoprecipitation with sequencing. https://www.activemotif.com/catalog/details/39901/ezh2-antibody-pab-1 "This EZH2 antibody was raised against a recombinant fusion protein corresponding to amino acids 1-370 of mouse EZH2; Applications validated by Active Motif: ChIP-seq: 5uL each"

Anti-H3K27me3 (Active Motif Cat#39155; RRID:AB_2561020) Indicated Applications: Immunohistochemsitry, Immunocytochemistry, Immunofluorescence, Western Blot, CUT&Tag, Chromatin Immunoprecipitation, Chromatin Immunoprecipitation with sequencing and others. https://www.activemotif.com/catalog/details/39155 "Applications validated by Active Motif: ChIP-seq: 5ug each; This antibody was raised against a peptide including trimethyl-lysine 27 of histone H3."

Anti-GFP (Thermo-Fisher #MS-1288-P0; RRID:AB_63267) Species Reactivity: Proteins from all species which are tagged with GFP. Previously used as a negative ChIP control in PMC4393352.

Anti-Rabbit IgG, HRP-linked (Novus Cat# NB7160; RRID:AB_10124655) Indicated Applications: Western Blot, ELISA, Immunocytochemsitry, Immunohistochemistry, Immunofluorescence https://www.novusbio.com/products/igg-h-l-antibody_nb7160
Please see PMCID: PMC8743034; PMC8743034 for prior use of this antibody for western blotting.

APC anti-mouse CD31 (Biolegend Cat# 102510; RRID:AB_312917) Indicated Application: Flow Cytometry https://www.biolegend.com/en-us/neuroscience-1/apc-anti-mouse-cd31-antibody-375 "Verified Reactivity: Mouse; FC Quality tested."
We previously used this antibody for cell sorting in PMCID: PMC8596110, and again validate it's utility with sorting followed by RNA-seq in this manuscript.

APC anti-mouse CD45 (Biolegend Cat# 103112; RRID:AB_312977) Indicated Application: Flow Cytometry https://www.biolegend.com/de-at/products/apc-anti-mouse-cd45-antibody-97 "Verified Reactivity: Mouse; FC Quality tested."
We previously used this antibody for cell sorting in PMCID: PMC8596110, and again validate it's utility with sorting followed by RNA-seq in this manuscript.

PE/Cy7 anti-mouse CD326/Ep-CAM (Biolegend Cat# 118216; RRID:AB_1236471) Application: Flow Cytometry https://www.biolegend.com/en-ie/search-results/pe-cyanine7-anti-mouse-cd326-ep-cam-antibody-5303 "Verified Reactivity: Mouse; FC Quality tested." We previously used this antibody for cell sorting in PMCID: PMC8596110, and again validate it's utility with sorting followed by RNA-seq in this manuscript.

Eukaryotic cell lines

Policy information about cell lines

Cell line source(s)	All the human cell lines were from Dr. Carla Kim's laboratory at Boston Children's Hospital and were from ATCC, expect BEAS-2B from Dr. Chengfeng Yang Lab also through ATCC, and HBEC-3KT from Dr. David Orren Lab from University of Kentucky, originally from Dr. John Minna UT Southwestern. All the primary murine cell lines were made from mouse models in our lab.

Authentication	All the human cell lines were authenticated by IDEXX BioAnalytics by CellCheck9 STR analysis.
Mycoplasma contamination	All cell lines used in this study were tested negative for mycoplasma contamination by Myco-Alert kit.
Commonly misidentified lines (See ICLAC register)	No misidentified lines were used.

Animals and other organisms

Policy information about studies involving animals; ARRIVE guidelines recommended for reporting animal research

Laboratory animals	Mouse.
Wild animals	No wild animals were used.
Field-collected samples	No field-collected samples were used.
Ethics oversight	All care and treatment of experimental animals were in accordance with Boston Children's Hospital and University of Kentucky institutional animal care and use committee (IACUC) guidelines.

Note that full information on the approval of the study protocol must also be provided in the manuscript.

Human research participants

Policy information about studies involving human research participants

Population characteristics	A total of 237 tissues were examined from 236 individuals. 98 were female and 138 were male. 9 were Black or African American and 227 were White. 162 were from Appalachian Kentucky. Ages at diagnosis ranged from 39 to 86 years old.
Recruitment	Not applicable, see below.
Ethics oversight	Leftover clinical specimens were obtained by BPTP with patient informed consent or waiver under an IRB approved protocol, and when transferred to our group, the samples had been de-identified and therefore exempt from further Institution Review Board approval.

Note that full information on the approval of the study protocol must also be provided in the manuscript.

ChIP-seq

Data deposition

- [x] Confirm that both raw and final processed data have been deposited in a public database such as GEO.
- [x] Confirm that you have deposited or provided access to graph files (e.g. BED files) for the called peaks.

Data access links<br>*May remain private before publication.*	The accession number is GSE182819 and the reviewer token is mlgtwkiaxzifxun
Files in database submission	GSM5537508 1_09X5_01CCUKY_WT_EZH2_mm10_i86<br>GSM5537509 2_09X6_01CCUKY_Het_EZH2_mm10_i87<br>GSM5537510 3_09X7_01CCUKY_WT_H3K27me3_mm10_i88<br>GSM5537511 4_09X8_01CCUKY_Het_H3K27me3_mm10_i91<br>GSM5537512 5_09X9_01CCUKY_Null-K27me3_H3K27me3_mm10_i92<br>GSM5537513 6_09XA_01CCUKY_WT-EPZ-K27me3_H3K27me3_mm10_i93<br>GSM5537514 7_09PH_01CCUKY_Pooled_Input_mm10_i84
Genome browser session (e.g. UCSC)	no longer applicable

Methodology

Replicates	Three biological replicates were pooled prior to ChIP
Sequencing depth	WT_EZH2: total reads = 32029907, unique alignment = 24241086, read length = 75bp, single-end;<br>Het_EZH2: total reads = 32463922, unique alignment = 25145726, read length = 75bp, single-end;<br>WT_H3K27me3: total reads = 32482162, unique alignment = 23847254, read length = 75bp, single-end;<br>Het_H3K27me3: total reads = 43333449, unique alignment = 29970820, read length = 75bp, single-end;<br>Null-K27me3_H3K27me3: total reads = 41142402, unique alignment = 18083074, read length = 75bp, single-end;<br>WT-EPZ-K27me3_H3K27me3: total reads = 31866213, unique alignment = 11155556, read length = 75bp, single-end;
Antibodies	EZH2 (Active Motif 39901) and H3K27me3 (Active Motif 39155)

Peak calling parameters	The SICER version 1.1 peak finding algorithm was used to identify regions of ChIP-seq enrichment over background, with window size 200bp, fragment size 200bp, gap size 600bp, and FDR value threshold of enrichment 1E-10 for all datasets.
Data quality	Aligned reads that had >2 mismatches and multimapping reads were removed followed by PCR deduplication. The resulting bam files were normalized to account for the differences in the sequencing depth. Genomic regions known to have low sequencing confidence were removed using blacklisted regions defined by the ENCODE project. Peaks with FDR < 1E-10 are selected for the downstream analysis.<br>The number of peaks with FDR < 0.05 and Fold-change >= 5 (reported by SICER) is:<br>WT_EZH2=215<br>Het_EZH2=135<br>WT_H3K27me3=310<br>Het_H3K27me3=625<br>Null-K27me3_H3K27me3=1437<br>WT-EPZ-K27me3_H3K27me3=1087
Software	Peak intervals were annotated using ChIPseeker to the nearest transcription start sites (TSS) using the KnownGene mm10 TSS annotation. Peak distribution patterns were obtained using seqplots across all merged intervals from −5 kb to +5 kb to include distal promoters and regulatory regions. The heatmaps are used for visualization of tag distributions which are mapped across target regions and were clustered into 4 groups based on the tag densities using k-means algorithm. The average values for all target regions in heatmaps were calculated and plotted in histograms. Peaks unique to each genotype or conserved in multiple genotypes were annotated by GREAT to associate each genomic region with all genes whose regulatory domain it overlaps. The resulting gene list was subjected to gene set enrichment analysis to identify significantly enriched gene signatures from GSEA curated signature gene sets.

Flow Cytometry

Plots

Confirm that:

- [x] The axis labels state the marker and fluorochrome used (e.g. CD4-FITC).
- [x] The axis scales are clearly visible. Include numbers along axes only for bottom left plot of group (a 'group' is an analysis of identical markers).
- [x] All plots are contour plots with outliers or pseudocolor plots.
- [x] A numerical value for number of cells or percentage (with statistics) is provided.

Methodology

Sample preparation	Tumor cell sorting: Tumors were dissected from the lungs of primary mice and tumor tissue was chop extremely finely with surgical scissors. Tumor chunks were incubated with 1mL 1x PBS with 60µL Collagenase/Dispase (Sigma, #10269638001) in a rotator at 37oC for 30min. After washed with 1X PBS, chunks were resuspended in 100µL 0.25% Trypsin-EDTA (Gibco) for 2min, then neutralized with 900µL PBS containing 10% FBS (PF10). Next, the digested tissue was filtered through a 100µm filter (VWR), then a 40µm filter (VWR) into a 50cc conical tube. Cell pellets were resuspended in 100µL Red Cell Lysis buffer (0.15 M NH4Cl, 10mM KHCO3, 0.1 mM EDTA, in 1L distilled H2O; filtered with 0.45 µm filter and stored at RT) for 2min at RT and added 800µL PF10. Single cell suspensions were stained using rat-anti-mouse antibodies including anti-mouse-EpCAM-PECy7 (BioLegend), anti-mouse-CD31-APC (BioLegend) and anti-mouse-CD45-APC (BioLegend). Live cells were gated by exclusion of 4',6-diamidino-2-phenylindole (DAPI) positive cells (Sigma, #D9542). All antibodies were incubated for 10-15 minutes at 1:100 dilutions.<br>Cell cycle analysis: Cells propagated in 2-dimensional dishes or 3-dimensional transwells were separated into single cells by 0.25% Trypsin. 1-10x105 cells were spun down in a 1.5mL microtube and resuspended in cold 300µL PF10. Then the resuspended cells were added dropwise to 700µL cold 70% Ethanol with slow vortexing and were incubated at 4°C for least 1 hour. Cells were pelleted by pulse spin and resuspended in 250µL/tube of 1mg/mL RNase A (Thermo Fisher, #EN0531) diluted in PBS for 30min. Cells were washed by 1ml PBS, pelleted and resuspended in 250µL/tube of 4µg/mL 7-Aminoactinomycin D (Invitrogen, #A1310).
Instrument	Sony iCyt with an 100µm nozzle; BD LSRII.
Software	ModFit LT software for cell cycle analysis.
Cell population abundance	Approximately 5,000-100,000 cells were collected for tumor sorting; 30,000 events/sample for cell cycle analysis.
Gating strategy	Cells were gated on DAPI-negative, then on FSC/SCC and single cells, then on CD45+/EpCAM-. A CD45+ single stained sample was used to determine the EpCAM gate.

- [x] Tick this box to confirm that a figure exemplifying the gating strategy is provided in the Supplementary Information.
